# Discrete Transforms and Matrix Rotation Based Cancelable Face and Fingerprint Recognition for Biometric Security Applications

**DOI:** 10.3390/e22121361

**Published:** 2020-11-30

**Authors:** Abeer D. Algarni, Ghada El Banby, Sahar Ismail, Walid El-Shafai, Fathi E. Abd El-Samie, Naglaa F. Soliman

**Affiliations:** 1 Department of Information Technology, College of Computer and Information Sciences, Princess Nourah Bint Abdulrahman University, Riyadh 84428, Saudi Arabia; adalqarni@pnu.edu.sa (A.D.A.); saismail@pnu.edu.sa (S.I.); nfsoliman@pnu.edu.sa (N.F.S.); 2 Department of Industrial Electronics and Control Engineering, Faculty of Electronic Engineering, Menoufia University, Menouf 32952, Egypt; ghada.elbanby@el-eng.menofia.edu.eg; 3 Electrical Engineering Department, Faculty of Engineering-Shoubra, Benha University, Cairo 11629, Egypt; 4 Department of Electronics and Electrical Communications Engineering, Faculty of Electronic Engineering, Menoufia University, Menouf 32952, Egypt; fathi_sayed@el-eng.menofia.edu.eg; 5 Department of Electronics and Communications, Faculty of Engineering, Zagazig University, Zagazig 44519, Egypt

**Keywords:** cancelable biometrics, discrete transforms, matrix rotation, DFT, DCT, FrFT, DWT

## Abstract

The security of information is necessary for the success of any system. So, there is a need to have a robust mechanism to ensure the verification of any person before allowing him to access the stored data. So, for purposes of increasing the security level and privacy of users against attacks, cancelable biometrics can be utilized. The principal objective of cancelable biometrics is to generate new distorted biometric templates to be stored in biometric databases instead of the original ones. This paper presents effective methods based on different discrete transforms, such as Discrete Fourier Transform (DFT), Fractional Fourier Transform (FrFT), Discrete Cosine Transform (DCT), and Discrete Wavelet Transform (DWT), in addition to matrix rotation to generate cancelable biometric templates, in order to meet revocability and prevent the restoration of the original templates from the generated cancelable ones. Rotated versions of the images are generated in either spatial or transform domains and added together to eliminate the ability to recover the original biometric templates. The cancelability performance is evaluated and tested through extensive simulation results for all proposed methods on a different face and fingerprint datasets. Low Equal Error Rate (EER) values with high AROC values reflect the efficiency of the proposed methods, especially those dependent on DCT and DFrFT. Moreover, a comparative study is performed to evaluate the proposed method with all transformations to select the best one from the security perspective. Furthermore, a comparative analysis is carried out to test the performance of the proposed schemes with the existing schemes. The obtained outcomes reveal the efficiency of the proposed cancelable biometric schemes by introducing an average AROC of 0.998, EER of 0.0023, FAR of 0.008, and FRR of 0.003.

## 1. Introduction

According to the evolving methods of hacking on biometric databases, there is a great need to develop non-traditional techniques to yield secure biometrics from the original ones that can be used for identifying the individuals with the ability to replace them with other alternatives in hacking scenarios [1,2,3,4,5]. To achieve a high level of security of biometric data, it should not be stored in its original raw format. Cancelable biometrics is an effective solution that guarantees template protection by generating renewable revocable templates to be stored in the database [6,7,8,9,10].

Cancelable biometric systems (CBSs) should provide high privacy and high security by utilizing different transformation methods in both enrolment and authentication stages [10,11,12,13,14,15,16]. Thus, the main objective of cancelable biometrics is to produce new deformed versions of original biometric templates to be stored in databases and used in the authentication stage. Several researchers have worked on generating robust cancelable biometrics schemes. In this section, we summarize these previously introduced CBSs. Choudhury et al. [17] proposed a cancelable iris recognition system based on the concept of steganography to generate transformed biometric templates. They exploited a combination of Huffman encoding and the DCT to obtain non-invertible transformation functions. Their system has its limitation of the need for a large number of images and more processing power. They achieved an Equal Error Rate (EER) of 1.2% and an acceptable area under the Receiver Operating Characteristic (ROC) curve. Wang et al. [18] introduced a combination of Discrete Fourier Transform (DFT) and Partial Hadamard Transform (PHT) to generate cancelable fingerprint templates. Their approach depends on representing the biometric data in binary format for simplicity of feature representation. They applied the PHT on the DFT of the binary biometric templates to get complex vectors that provide a high security level of the original binary vectors to prevent their restoration. They tested their proposed system on three datasets, and achieved EERs of 1% for FVC2002DB1, 2% for FVC2002DB2, and 5.2% for FVC2002DB3. Soliman et al. [19] introduced two different cancelable approaches for iris and face biometric images. These approaches adopted feature vectors for the biometrics to be encrypted by the Double Random Phase Encoding (DRPE) algorithm. They exploited the Scale-Invariant Feature Transform (SIFT) and the Gabor filter to generate the feature matrix. Their approach achieved an EER of 0.17% and an AROC of 99.3%. 

Kaur and Khanna [20] proposed a multi-level transform biometric template protection technique based on generating new distorted versions of the original biometrics using random projection followed by applying Log-Gabor transform to get both Log-Gabor magnitude and phase to be XORed with a Random Grid (RG) to give an encrypted template for each scale and orientation. The next step is to subject the encrypted template pattern to a non-linear median filter. The output of this step is then normalized, reshaped, and re-sampled to generate the final protected transformed biometric. Umer et al. [21] presented a cancelable iris recognition system that begins with localization and normalization of the iris object followed by applying the SIFT to extract dense SIFT descriptors. K-means clustering algorithm is applied on the collection of descriptors to form a dictionary pattern upon which sparse representation coding and spatial pyramid mapping are performed. The final step is to apply a modified bio-hashing technique to generate cancelable features to be stored in the database. The same sequence is applied in the authentication stage to test new templates against stored encrypted iris features to perform the iris recognition process. This system has been tested using six benchmark iris databases.

Yang et al. [22] presented a multi-biometric security system for fingerprint and finger vein biometrics based on combining different features extracted from these two biometrics. They used a minutiae-based technique to extract features of fingerprints and an image-based technique to extract finger vein features. The output feature vector produced for each biometric is subjected to a binary data conversion process to give binary feature vectors for both fingerprints and finger veins. They suggested three different fusion methods based on Enhanced Partial Discrete Fourier Transform (EP-DFT) to give a final feature vector to be stored in the database in the enrolment phase. A similar technique is implemented in the verification phase to give the query transformation feature vector based on EP-DFT. A similarity score is estimated to test the matching between the stored features and query actual features. This method achieved a high security level with a minimum EER of 0.12%. The second fusion method depends on the transformation of the two biometric features by EP-DFT, followed by the concatenation of the two output feature vectors, to give a single binary vector. 

A random distance technique was proposed by Kaur and Khanna to generate cancelable biometric templates [23]. They applied this technique on different modalities. It meets important requirements of revocability and non-invertability. Cancelable fingerprint patterns are generated based on fusing structures at the feature level. Local and distant structures are computed from minutiae points of fingerprints to generate fused bit strings. In the next step, DFT is applied to get the cancelable fingerprint templates. This technique achieved an EER of 1.6% on the FVC 2002 DB2 database and an EER of 12.7% on the FVC 2004 DB2 database.

Choudhary and Naik [24] presented an overview of multiple biometrics used for authentication. They focused on challenges in multimodality biometrics by using fusion with different levels and different techniques. They discussed the factors to be considered to build a robust authentication system based on multimodality biometrics, such as the selection of the biometrics to be combined, the level of fusion, the effect of using multimodality biometrics on complexity and processing time, and the required storage space needed for the secure templates. They discussed also the concept of the integration of multimodality biometrics in order to generate a secure template and found that there is no single method that can be utilized with all biometrics. 

Patil et al. [25] proposed a secret sharing and Radio Frequency Identification (RFID) scheme for biometric authentication. They succeeded in generating secure templates and storing them in the database. Their proposed approach can be extended to include multimodality biometrics. They achieved low computational complexity, high reliability, and high security. Namrata et al. [26] presented a biometric authentication system to verify the identity of the user. Their proposed model uses the orientation values from a fingerprint and the minutiae from another fingerprint to generate the combined template in the enrollment phase. For the authentication phase, the stored encrypted template will be used to decrypt the OTP to complete the electronic financial transaction. They worked with the most common evaluation metrics for authentication processes including False Match Rate (FMR), False Acceptance Rate (FAR), False Non-Match Rate (FNMR), False Rejection Rate (FRR), Failure to Acquire Rate (FTA), and Equal Error Rate (EER). Abou elazm et al. [27] proposed a cancelable face and fingerprint recognition scheme based on the 3D jigsaw transform and optical encryption. Their scheme exploits the Fractional Fourier Transform (FRFT) to generate encrypted biometric templates. They verified the efficiency of their proposed scheme against other traditional cryptosystems by computing the EER, the FAR, the FRR, and the AROC. They achieved an AROC value of 0.9997 with an EER of 9.3997 × 10^−15^. 

Abdelatif et al. presented an approach for cancelable face recognition that depends on Convolutional Neural Networks (CNNs) [28,29]. Its idea begins by isolating the face, eyes, nose, and mouth regions. A CNN is designed for each region to extract deep features. The deep feature vectors are fused together for size preservation, and hence, a bio-convolving process is implemented to encrypt the extracted feature vector through a convolution process with a random mask. This approach presents good performance in the verification process. Unfortunately, it can be hacked if the convolution mask is known and a strong deconvolution algorithm is used. Abdelatif et al. developed their approach by incorporating hand-crafted features with their deep features [30]. They extract hand-crafted features from both face and iris images and apply a dimensionality reduction stage based on PCA to create features suitable in length to the deep features. Feature fusion is adopted in the last stage to generate a common feature vector that is encrypted through bio-convolution. Although the hand-crafted features add mode information to the recognition process, the bio-convoluting is still the weak point of this approach. 

Soliman et al. investigated the utilization of different chaotic maps for encrypting iris image feature vectors [31]. Both logistic and modified logistic maps have been considered for this issue. A correlation-based approach has been used in the verification process of encrypted iris vectors. This approach achieved a good performance as a cancelable biometric recognition system, but its drawback is the weak encryption algorithm based on chaotic maps only. 

Hashad et al. presented a cryptosystem that can be used for cancelable fingerprint recognition [32]. Its idea is based on a pre-processing step. This step is merely a fusion process between fingerprint images with few details and auxiliary images that are rich in details. The fusion masks the main discriminative patters of the fingerprints. After that, encryption is performed with chaotic maps to generate the cancelable masks. This approach succeeds in securing the fingerprints, but the encryption is not strong enough. Away from the encryption process to generate cancelable masks, Soliman et al. presented an approach for generating cancelable masks that depends on intended distortion of the biometric traits [33]. This approach depends on the utilization of the comb filter as a multi-band filter with several nulls to distort the iris feature vector. The comb filter is non-invertible as it has several nulls in its magnitude spectrum. This approach succeeded in giving high performance of the cancelable iris recognition system.

Algarani et al. worked on the topic of cancelable face recognition by adopting pre-processing of the biometric traits first [34]. Two trends have been presented in this paper. The first one depends on fuzzy logic to modify the image dynamic range and the second one depends on homomorphic decomposition of the images to isolate the more informative reflectivity components. After that, encryption with random projection is performed. The random projection encryption is applied on signatures extracted from the biometric traits. These signatures are not invertible. Hence, the merit of encryption here is to enhance the security, while keeping irreversibility. 

Cherrat et al. [35] presented a hybrid system for multi-biometrics based on CNNs for feature extraction. They combine fingerprint, finger vein, and face biometric images after pre-processing and feature extraction, separately. For the fingerprint recognition, three processes are applied; pre-processing to extract the foreground and background regions, feature extraction based on CNNs, and classification based on a SoftMax layer. For the finger vein recognition, enhancement is performed based on image fusion using CNNs for feature extraction, and Random forest is conducted for the classification. Finally, the outputs of the three systems are fused according to a pre-determined score to improve the identification of biometrics. Hui Xua [36] presented a multi-modal biometric system based on a CNN for combining face, iris, and palm print. They studied the effect of changing the number of layers on the recognition process. The simulation results proved that fusion based on two layers improves the recognition accuracy.

All the above-mentioned approaches have their own characteristics and limitations. This work is concerned with designing transformation methods to achieve sufficient distortion to be applied to all original biometric templates stored in the database in order to make them more complex and difficult to be inverted or identified. Therefore, the principal goal of this paper is to introduce an efficient method to generate cancelable biometric templates based on discrete transforms and matrix rotation. The rationale behind the utilization of discrete transforms, such as DCT, DFT, and DFrFT, is to perform some sort of data diffusion. Unfortunately, these transforms are invertible, and hence they are inappropriate for cancelable biometric applications. So, we suggest the utilization of matrix rotation in a transform domain and the addition of rotated versions. If an addition is carried out on the rotated versions, the reconstruction of the original transform domain matrix becomes impossible. Hence, if the transform inversion is implemented, the original biometric template is not recoverable. Cancelability is guaranteed through the variability of the rotation angles prior to the addition process. The DWT has another basis of operation, which is the sub-band decomposition. It is also investigated in this paper as a discrete transform to be implemented prior to the rotation process.

This article is structured as follows: Section 2 presents the basics of the related concepts used in this paper. The proposed cancelable biometric systems (CBSs) based on matrix rotation in discrete transform domains are introduced in Section 3. Section 4 provides the simulation outcomes and the comparison analyses to evaluate the performance of the suggested CBSs. Section 5 provides concluding remarks and some directions for future research.

## 2. Preliminaries 

This section presents the main basic concepts of the matrix transformation methods used to generate the deformed versions of the original biometric templates. 

### 2.1. Basics of the DFT

Fourier Transform is considered as an important conversion technique due to its wide range of applications. It has been introduced by Baptiste Fourier (1768–1830). Images in Fourier transform are decomposed into sine and cosine components. The two-dimensional Discrete Fourier Transform (DFT) is given by the following relation [37]:

(1) $$F\left(k,l\right)=\overset{N-1}{\underset{i=0}{\sum }}\overset{M-1}{\underset{j=0}{\sum }}f\left(i,j\right)e^{-2i\pi \left(\frac{ki}{N}+\frac{lj}{M}\right)}$$

where *f*(*i*,*j*) is the value of pixel intensity at (*i*,*j*) in the image of size *N* × *M* in the spatial domain. Generally, DFT uses complex mathematical relations that consume more computational time in processing. 

### 2.2. Basics of the DFrFT

The DFrFT can be considered as the generalized configuration of the classical Fourier transform. It provides a more flexible frequency distribution than the conventional DFT [38,39]. Further security can be obtained for the system by adding another parameter called “α”. It is known that the Fourier transform is performed through the rotation of any signal by an angle of *π*/2 in the time-frequency plane. On the other hand, the DFrFT eliminates that limit and permits rotation with any angle ‘*α*’, which is not required to be a multiple of *π*/2. The DFrFT is similar to the ordinary Fourier transform when *α* = 1. The DFrFT was expressed in [38] for a signal *f* (*t*) for order ‘*α*’ as follows:

(2) $$F_{p}\left(u,t\right)=\;\overset{\infty }{\underset{-\infty }{\int }}f\left(t\right)K_{p}\left(u,t\right)dt$$

where *K_p_* is the kernel defined as follows:

(3) $$K_{p}(u,t)=\left\{\begin{array}{cc} \sqrt{\frac{1-jcot\alpha }{2\pi }}\exp\left(j\frac{t^{2}+u^{2}}{2}\cot\alpha -jut\;cse\;\alpha \right) & \alpha \neq n\pi \\ \delta (t-u) & \alpha =2n\pi \\ \delta (t+u) & \alpha =(2n\pm \pi ) \end{array}\right\}$$

### 2.3. Basics of the DCT

The DCT divides the image into different bands represented as a low-frequency band, a mid-frequency band, and a high-frequency band. The 2D-DCT can be represented as follows [40]:

(4) $$F\left(k,l\right)=a\left(k\right)\cdot a\left(l\right)\overset{N-1}{\underset{i=0}{\sum }}\overset{M-1}{\underset{j=0}{\sum }}f\left(i,j\right)\cdot cos\left[\frac{\left(2i+1\right)k\pi }{2N}\right]cos\left[\frac{\left(2j+1\right)l\pi }{2M}\right]$$

where $a\left(k\right)=\{\begin{array}{cc} \sqrt{\frac{1}{N}} & k=0\\ \sqrt{\frac{2}{N}} & k=1,\;2,\;\ldots \ldots .N-1 \end{array}$ and $a\left(l\right)=\{\begin{array}{cc} \sqrt{\frac{1}{M}} & l=0\\ \sqrt{\frac{2}{M}} & l=1,\;2,\;\ldots \ldots .N-1 \end{array}$.

### 2.4. Basics of the DWT

This transform maps the biometric images into the wavelet domain based on separating and decomposing the intensity values in images into a low-frequency band and a high-frequency band. The DWT is considered as a highly effective tool to be used in a wide range of applications in image processing. It decomposes the image into four blocks, normally labeled as LL, HL, LH, and HH [41,42,43].

The 2D-DWT can be represented with a 2D-scaling function φ(x,y) and three 2D wavelet functions: $\psi^{H}\left(x,y\right),\;\psi^{V}\left(x,y\right),\;\mathrm{and}\;\psi^{D}\left(x,y\right),\;$where each $\psi {\left(.\right)}^{}$ function measures variations along with horizontal, vertical, and diagonal directions. The DWT can be represented as:

(5) $$W_{\emptyset }\left(j_{0},m,n\right)=\frac{1}{\sqrt{NM}}\overset{N-1}{\underset{x=0}{\sum }}\overset{M-1}{\underset{y=0}{\sum }}f\left(x,y\right)\emptyset_{j_{0},m,n}\left(x,y\right)$$

(6) $$W_{\psi }^{i}\left(j,m,n\right)=\frac{1}{\sqrt{NM}}\overset{N-1}{\underset{x=0}{\sum }}\overset{M-1}{\underset{y=0}{\sum }}f\left(x,y\right)\Psi_{j,m,n}^{i}\left(x,y\right)$$

The scaling and translated functions are represented as follows:

(7) $$\emptyset_{j,m,n}\left(x,y\right)=2^{j/2}\emptyset \left(2^{j}x-m,\;2^{j}y-n\right)$$

(8) $$\psi_{j,m,n}^{i}\left(x,y\right)=2^{j/2}\psi^{i}\left(2^{j}x-m,\;2^{j}y-n\right)$$

where *m* and *n* are the translation quantities i∈(H,V,D).

### 2.5. Basics of Matrix Rotation

**Figure 1:**
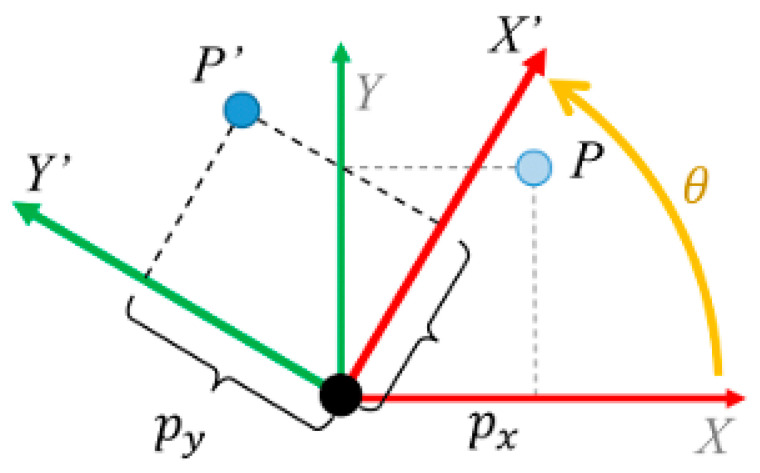
Matrix rotation with an angle θ.

The basic concept of rotating a two-dimensional image by an angle α as shown in Figure 1 is clarified in the following discussion [44,45]. A pixel at point *P* with spatial coordinates (x,y) in the original image can be rotated to new spatial coordinates $\left(x^{’},y^{’}\right)$, hence,

(9) $$x^{,}=x\cdot cos\alpha -y\cdot sin\alpha$$

(10) $$y^{,}=x\cdot sin\alpha +y\cdot cos\alpha$$

Based on the previous relations, the original pixels of an image can be rotated by different angles in a counter-clockwise direction or in a clockwise direction according to the sign of the rotation angle α. 

## 3. Proposed CBS Systems Based on Matrix Rotation in Discrete Transform Domains

In this section, we present an enhanced level of security for human biometric recognition. Our study depends on the generation of new revocable templates depending on adopting a bank of matrix rotations with different selected rotation angles combined with a matrix transformation method to meet cancelable biometrics requirements and achieve a high level of security and user privacy.

### 3.1. Proposed Bank of Rotations with DWT

**Figure 2:**
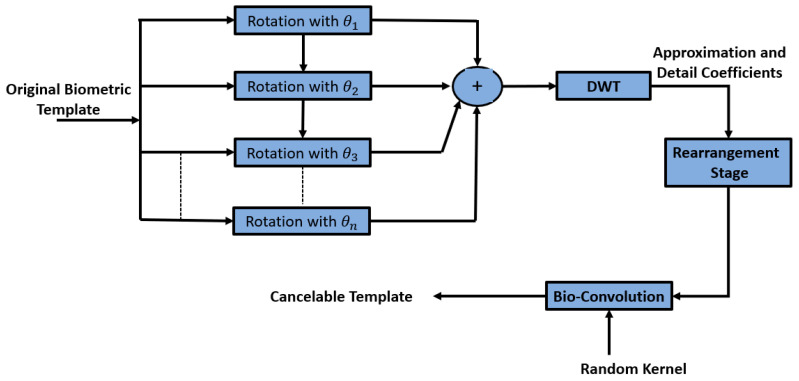
Proposed method based on a bank of rotations and DWT to generate the cancelable biometric templates.

The first proposed method is depicted in Figure 2 and in Algorithm 1, and it depends on the Wavelet-Based Bank of Rotations (WBBOR). It offers three degrees of freedom to ensure high efficiency. The first degree depends on pixel rotation from different angles. The second degree is represented in detailed rotated coefficients extracted from DWT and rearranged to form the second stage of biometric distortion. The final degree is represented in the bio-convolution based on a random kernel to produce high-level biometric distortion.

**Algorithm 1** The pseudo code of the Wavelet-Based Bank of Rotations (WBBOR) method
  1:**Input:** Biometric image I(i,j).  2:**Output:** Distorted image $I_{S}\left(i,j\right).$  3:**Step 1.** Pre-processing adjustment is performed on each biometric image.  4:**Step 2.** Image rotation is applied with different rotation angles, $\theta_{n}$, where the total rotated image is computed by:  5:
(11)$$I_{rtotal}\left(i,j\right)=\sum (I\left(i,j\right),\theta_{n})$$
  6:**Step 3.** (a) DWT is applied on the rotated image by Equation (3).  7:(b) Components are rearranged to form:  8:
(12)$$I_{H}\left(i,j\right)=\left[LL\;LH;HL\;HH\right]$$
  9:**Step 4.** Bio-convolution is applied to generate the final encrypted biometric image by:  10:
(13)$$I_{S}=I_{H}⊗\;R_{kernal}$$



The addition of multiple rotated versions of the biometric images guarantees some sort of distortion that is not reversible. The output of this stage is decomposed with wavelet decomposition into sub-bands, which are rearranged in an optional manner. Finally, encryption is performed as a last stage of security to secure the biometric templates. In cases of hacking, it is possible to change the rotation angles or the number of rotations. In addition, it is possible also to change the arrangement after DWT, the encryption algorithm or the key.

### 3.2. Proposed Bank of Rotations Based on DCT

**Figure 3:**
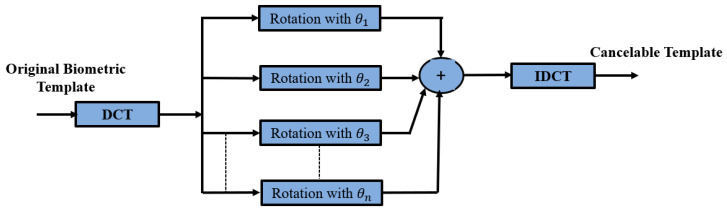
Proposed method based on DCT and a bank of rotations to generate the cancelable biometric templates.

The templates of cancelable biometrics are generated by the transformation of biometric templates with DCT followed by matrix rotation of the generated coefficients, as illustrated in Figure 3. The DCT transform gives some sort of data diffusion, but it is invertible. Moreover, matrix rotation is applied with different rotation angles. The addition of rotated versions gives more distortion of the data in the DCT domain. The addition of rotated versions is not invertible to obtain a high level of security.

In the proposed Bank of Rotations Based on the DCT (BRBDCT) method, as depicted in Algorithm 2, the DCT is applied on the raw biometric gray-scale image to generate an image in the DCT domain, which can be represented as I_dct [*N* × *N*]. Secondly, the DCT image is rotated with different angles ($\theta_{n1},\;\theta_{2},\;\theta_{3},\;and\;\theta_{n4}$). The four outputs are added together to generate a new image in the DCT domain. Finally, a secure template is generated using the Inverse DCT (IDCT). This template is stored in the database and the same process is performed in the authentication.
**Algorithm 2** The pseudo code of the Bank of Rotations Based on the DCT (BRBDCT) method  1:**Input:** Biometric image I(i,j).  2:**Output:** Distorted image $I_{S}\left(i,j\right).$  3:**Step 1.** A pre-processing adjustment is performed on each biometric image.  4:**Step 2.** The DCT is applied to produce the DCT coefficients using Equation (2).  5:**Step 3.** Image rotation is performed with different angles of rotation $\theta_{n}$ for the DCT coefficients, while the total distorted image with rotation is computed by Equation (9).  6:**Step 4.** The deformed biometric template is obtained by employing the IDCT.

### 3.3. Proposed Bank of Rotations Based on FFT or FrFT

**Figure 4:**
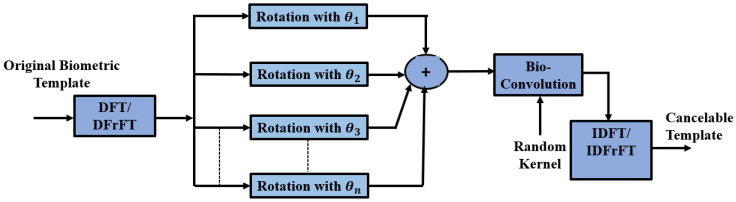
Proposed method based on DFT or DFrFT and a bank of rotations to generate the cancelable biometric templates.

In this subsection, a proposed method based on the rotation of biometrics in the frequency domain is introduced to enhance the security of biometric templates as shown in Figure 4 and Algorithm 3. The Fourier transform allows us to represent an image by its frequency spectrum. Rotation and addition are performed on complex values leading to distorted complex patterns of biometrics. The added complex patterns are very difficult to recover.

To increase the security, the summation of the rotations is bio-convolved with a random kernel with the same size as the original template. Finally, the inverse FFT is applied to the output of the bio-convolution. This transformation can produce irreversible deformed patterns. More security is added to the system using the DFrFT to exploit its characteristics based on its inherent rotation angle.
**Algorithm 3** The pseudo code of the Bank of Rotations Based on FFT (BRBFFT) method  1:**Input:** Biometric image I(i,j).  2:**Output:** Distorted image $I_{S}\left(i,j\right).$  3:**Step 1.** A pre-processing adjustment is performed on each biometric image.  4:**Step 2.** Apply the DFT/DFrFT transformation to produce the frequency representation of the template using Equations (1) or (2).  5:**Step 3.** Image rotation is applied with different angles $\theta_{n}$ for the DCT coefficients, while the total rotated image is computed by Equation (11).  6:**Step 4.** Bio-convolution is applied.  7:**Step 5.** Construction of the deformed biometric template is implemented by employing the inverse DFT/DFrFT.

## 4. Performance Evaluation and Test Results 

Any biometric system is composed of two stages: the enrollment stage and the verification stage. The ultimate aim of cancelable biometric systems is that in the enrollment stage, the original biometric templates are converted into different forms by using non-invertible transformation functions. In the verification stage, query data are subjected to the same non-invertible transformations for matching. 

In the suggested cancelable biometric technique, we employ four different transforms—DWT, DCT, DFT, and DFrFT—that have different characteristics to investigate the performance in spatial and transform domains. The suggested cancelable methods are composed of two different parts: the transformation which induces some confusion in the data and the bank of rotations to induce more distortion. Therefore, retrieving the raw template biometric data is infeasible and computationally difficult. 

**Figure 5:**
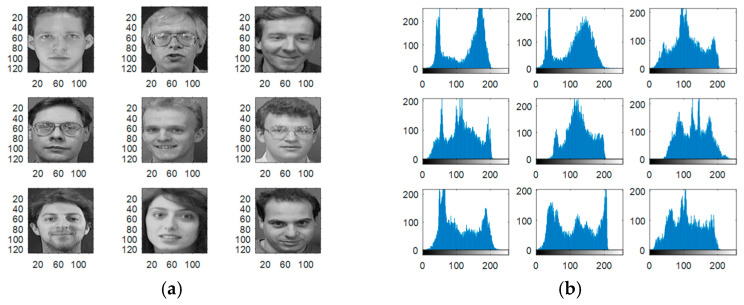
Samples of ORL database faces used as original biometrics and their histograms. (**a**) Original biometrics; (**b**) Biometrics histograms.

**Figure 6:**
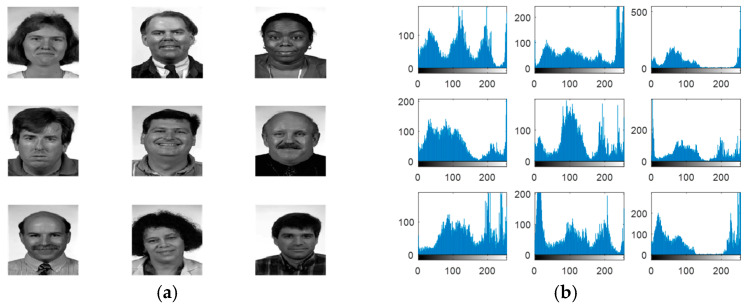
Samples of FERET database faces used as original biometrics and their histograms. (**a**) Original biometrics; (**b**) Biometrics histograms.

**Figure 7:**
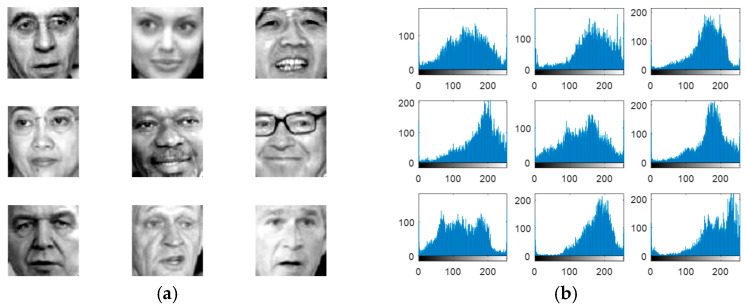
Samples of LFW database faces used as original biometrics and their histograms. (**a**) Original biometrics; (**b**) Biometrics histograms.

**Figure 8:**
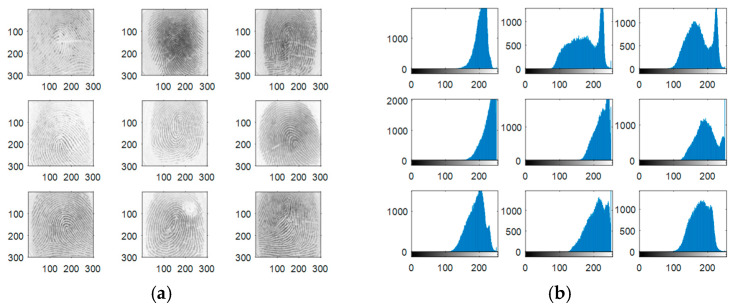
Samples of the first fingerprints database used as original biometrics and their histograms. (**a**) Original biometrics; (**b**) Biometrics histograms.

**Figure 9:**
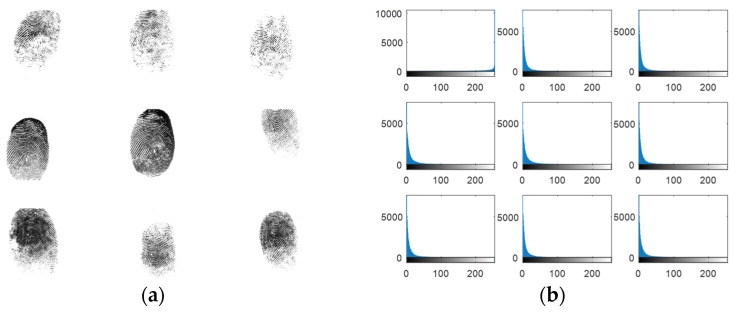
Samples of the second fingerprints database used as original biometrics and their histograms. (**a**) Original biometrics; (**b**) Biometrics histograms.

Experiments are carried out on the proposed methods to investigate and evaluate their performance using five different standard datasets: three different face datasets and two different fingerprint datasets. The tested facial images used in our simulations and evaluations are obtained from the Research Laboratory for Olivetti and Oracle (ORL) database [46], the NiST Face Recognition Technology (FERET) dataset [47], and the Mass Labelled Faces in the Wild (LFW) dataset of the University of Massachusetts’ Computer Vision laboratory [48]. The fingerprint images used in our simulations and evaluations are obtained from [49,50]. In order to ensure the validation of the proposed cancelable methods for both face and fingerprint recognition, we worked on 20 different samples of images from each of the face databases and 20 different samples of images from the fingerprint databases. These face and fingerprint images were chosen randomly. The samples of the face images and their histograms are illustrated in Figure 5, Figure 6 and Figure 7. The samples for the fingerprint images are illustrated in Figure 8 and Figure 9.

The obtained simulation results are evaluated depending on False Acceptance Rate (FAR), False Rejection Rate (FRR), and Equal Error Rate (EER). In addition, histograms of encrypted images, correlation scores for genuine and imposter patterns of biometric images, Probability Distribution Functions (PDFs) for genuine and imposter distributions, and ROC curves are used for evaluation.

The FAR of the system can be defined as the times an impostor accesses the system as a genuine user, which reflects the robustness of the system against a zero-effort attack. On the other side, the FRR denotes the times the system rejects genuine user access. The point at which the value of the FAR and FRR are equal is called “EER”. When this value is smaller, the performance of the biometric system is better.

In order to ensure the effectiveness of the matrix rotations, we will apply the rotation in the following cases:

1. Rotation in the spatial domain.
2. Rotation followed by DWT as depicted in Section 3.1.
3. Rotation in the frequency domain using DCT as explained in Section 3.2.
4. Rotation in the frequency domain using FFT as explained in Section 3.3.
5. Rotation in the FrFT domain in two different scenarios to select the best performance.

**Figure 10:**
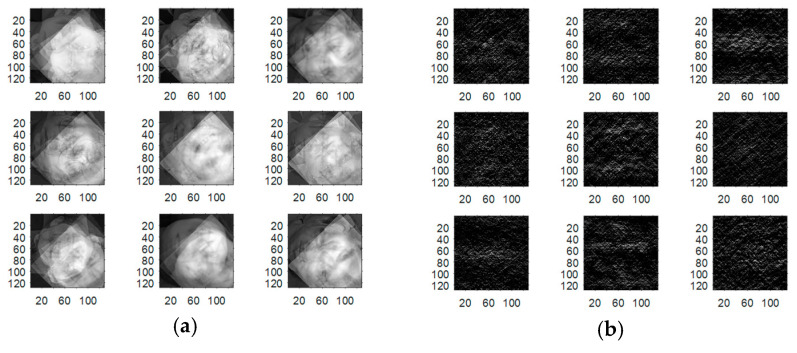

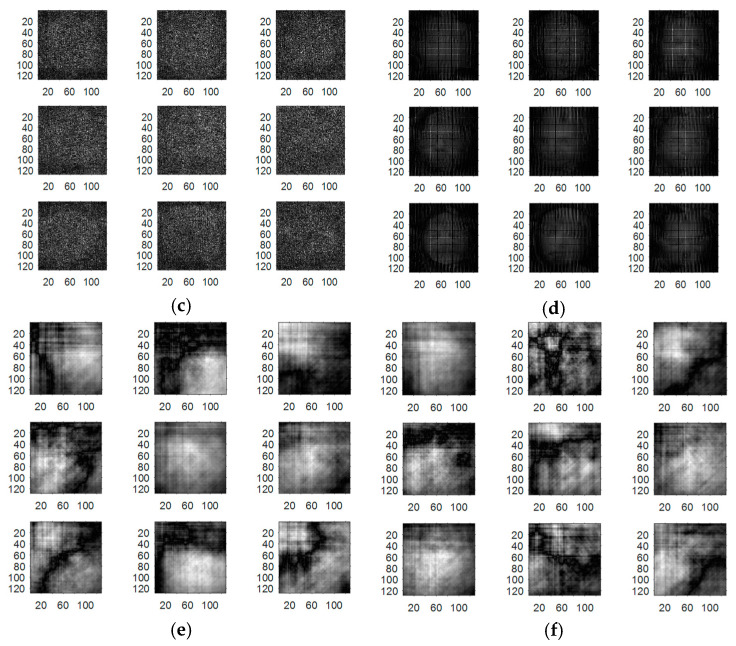
Encrypted ORL biometric faces. (**a**) Rotation in spatial domain; (**b**) Rotation followed by DWT; (**c**) Rotation in FFT domain; (**d**) Rotation in DCT domain; (**e**) Rotation in FrFT domain [90, 90]; (**f**) Rotation in FrFT domain [370, 370].

**Figure 11:**
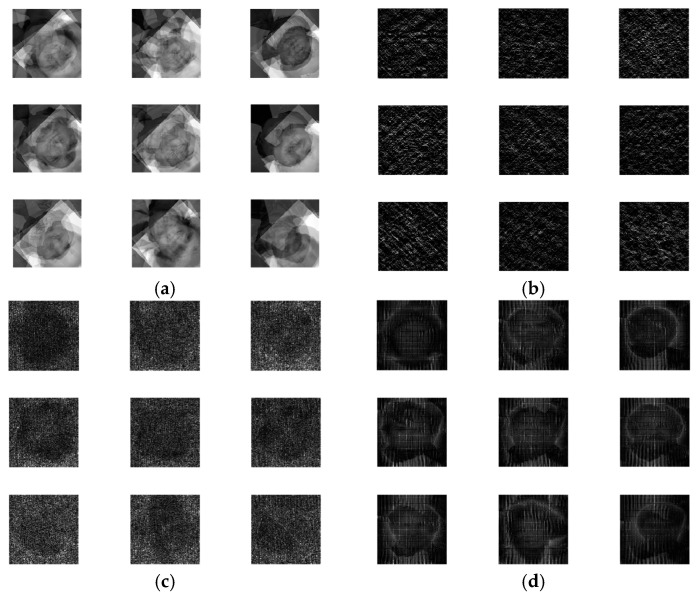

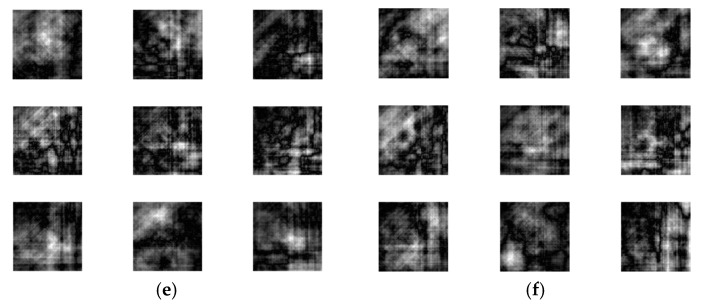
Encrypted FERET biometric faces. (**a**) Rotation in spatial domain; (**b**) Rotation followed by DWT; (**c**) Rotation in FFT domain; (**d**) Rotation in DCT domain; (**e**) Rotation in FrFT domain [90, 90]; (**f**) Rotation in FrFT domain [370, 370].

**Figure 12:**
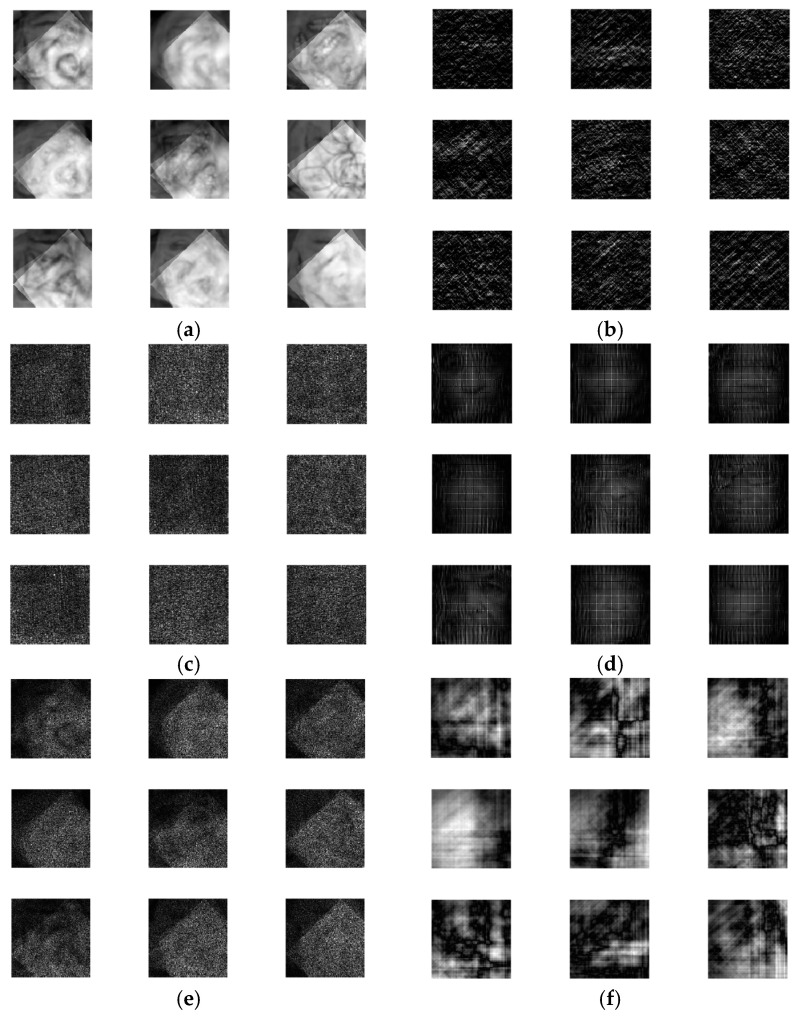
Encrypted LFW biometric faces. (**a**) Rotation in spatial domain; (**b**) Rotation followed by DWT; (c) Rotation in FFT domain; (**d**) Rotation in DCT domain; (**e**) Rotation in FrFT domain [90, 90]; (**f**) Rotation in FrFT domain [370, 370].

**Figure 13:**
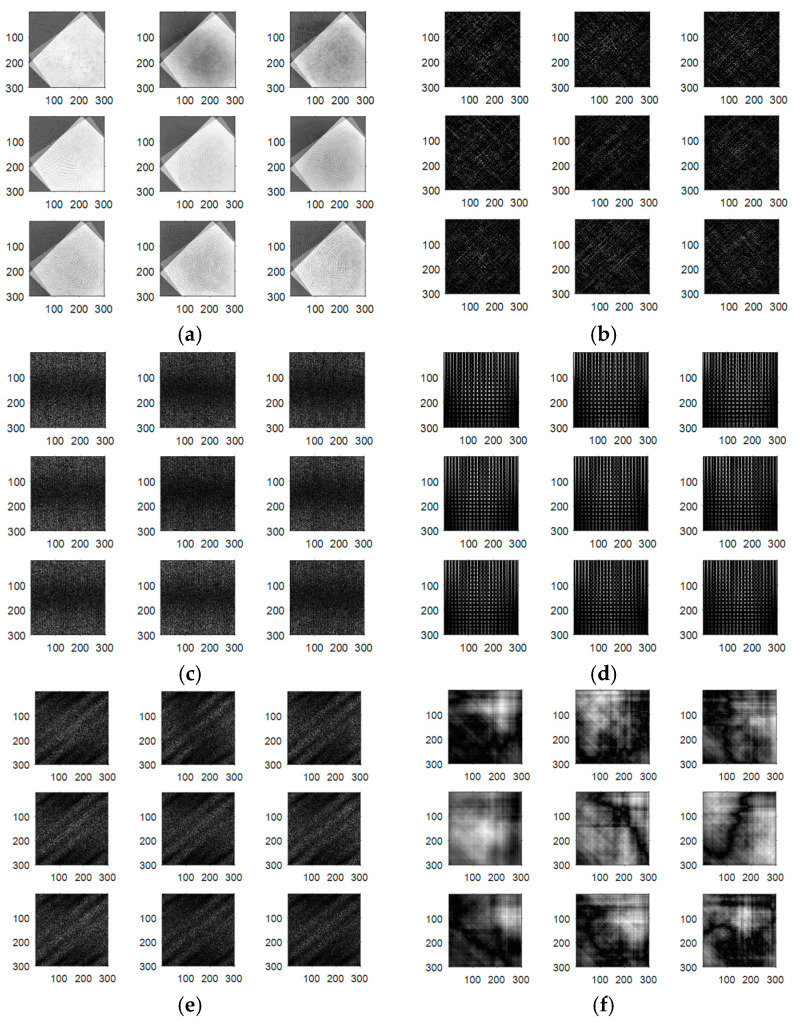
Encrypted first biometric fingerprints. (**a**) Rotation in spatial domain; (**b**) Rotation followed by DWT; (**c**) Rotation in FFT domain; (**d**) Rotation in DCT domain; (**e**) Rotation in FrFT domain [45, 45]; (**f**) Rotation in FrFT domain [180, 90].

**Figure 14:**
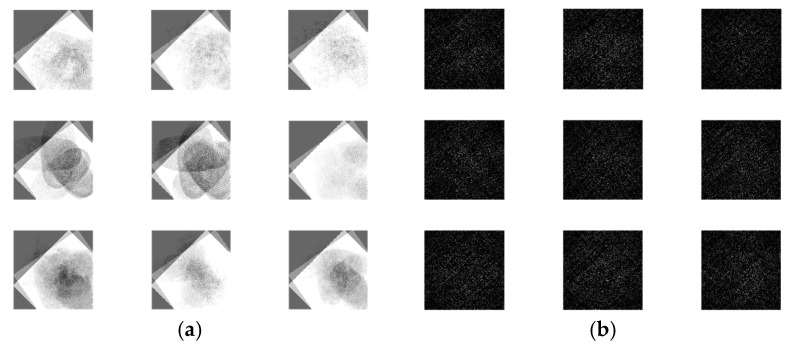

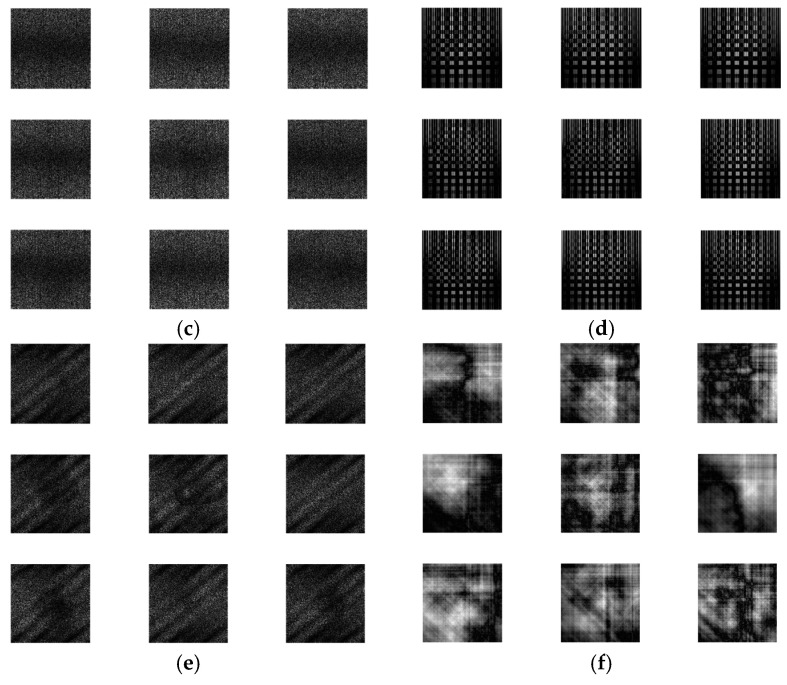
Encrypted second biometric fingerprints. (**a**) Rotation in spatial domain; (**b**) Rotation followed by DWT; (**c**) Rotation in FFT domain; (**d**) Rotation in DCT domain; (**e**) Rotation in FrFT domain [45, 45]; (**f**) Rotation in FrFT domain [180, 90].

**Figure 15:**
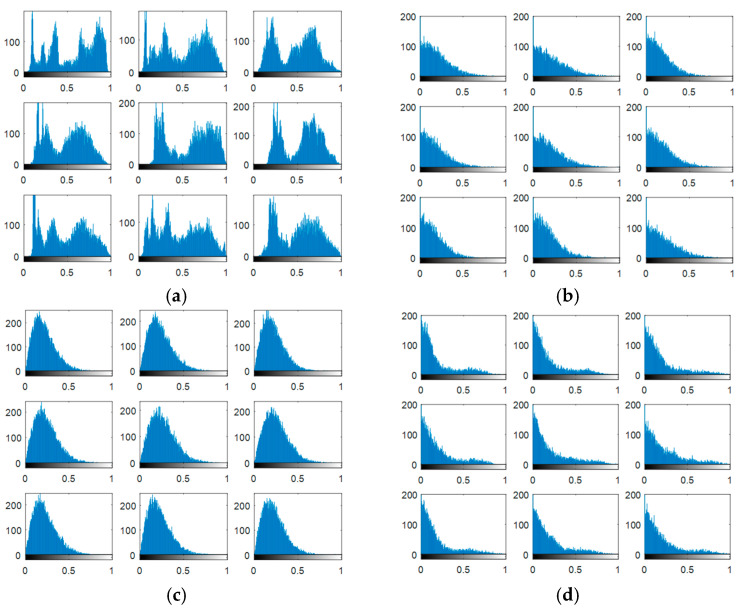

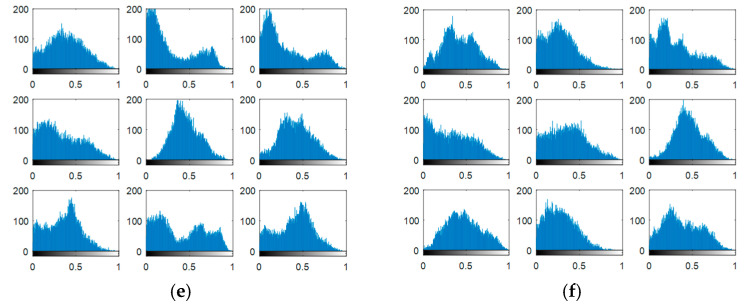
Histogram of encrypted images for ORL faces biometrics. (**a**) Rotation in spatial domain; (**b**) Rotation followed by DWT; (**c**) Rotation in FFT domain; (**d**) Rotation in DCT domain; (**e**) Rotation in FrFT domain [90, 90]; (**f**) Rotation in FrFT domain [370, 370].

**Figure 16:**
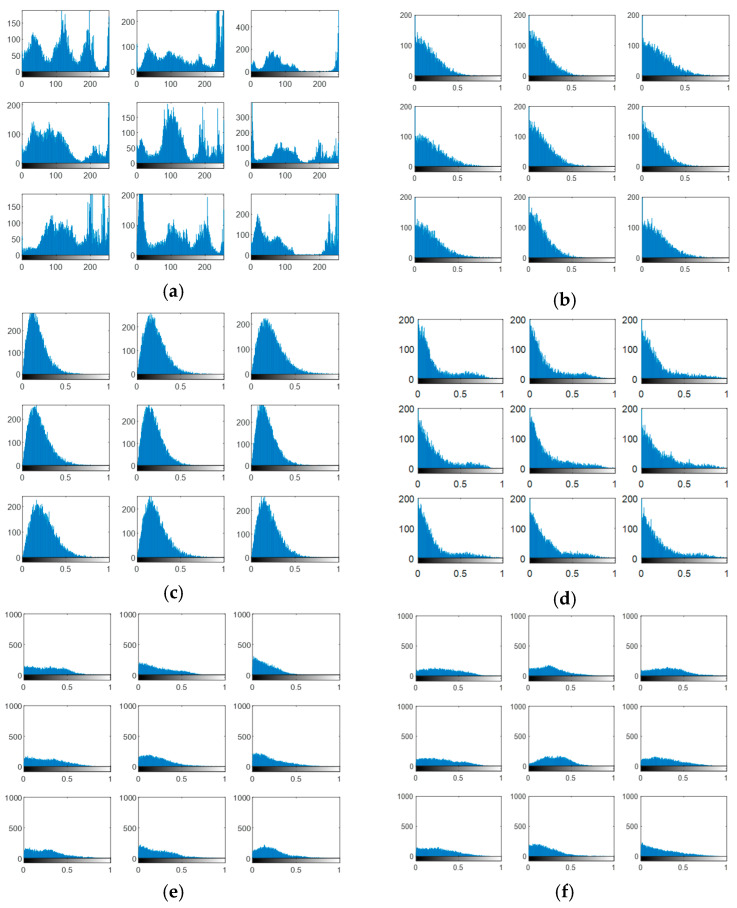
Histogram of encrypted images for FERET faces biometrics. (**a**) Rotation in spatial domain; (**b**) Rotation followed by DWT; (**c**) Rotation in FFT domain; (**d**) Rotation in DCT domain; (**e**) Rotation in FrFT domain [90, 90]; (**f**) Rotation in FrFT domain [370, 370].

**Figure 17:**
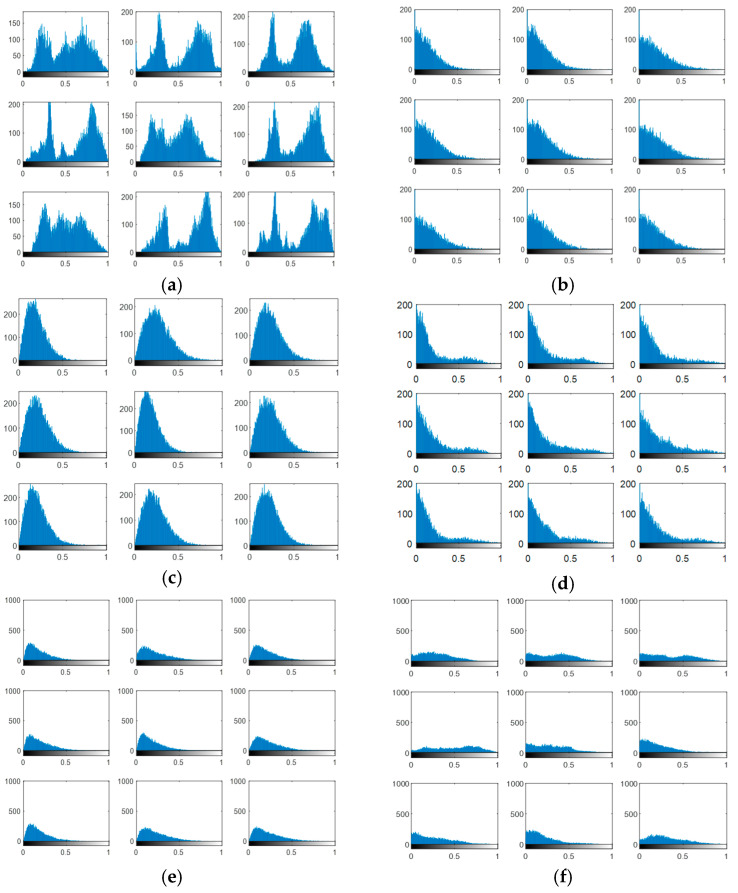
Histogram of encrypted images for LFW faces biometrics. (**a**) Rotation in spatial domain; (**b**) Rotation followed by DWT; (**c**) Rotation in FFT domain; (**d**) Rotation in DCT domain; (**e**) Rotation in FrFT domain [90, 90]; (**f**) Rotation in FrFT domain [370, 370].

**Figure 18:**
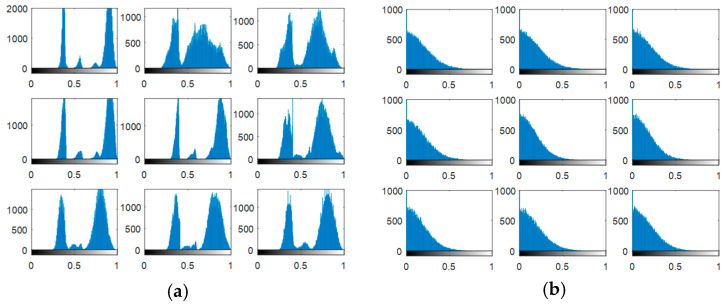

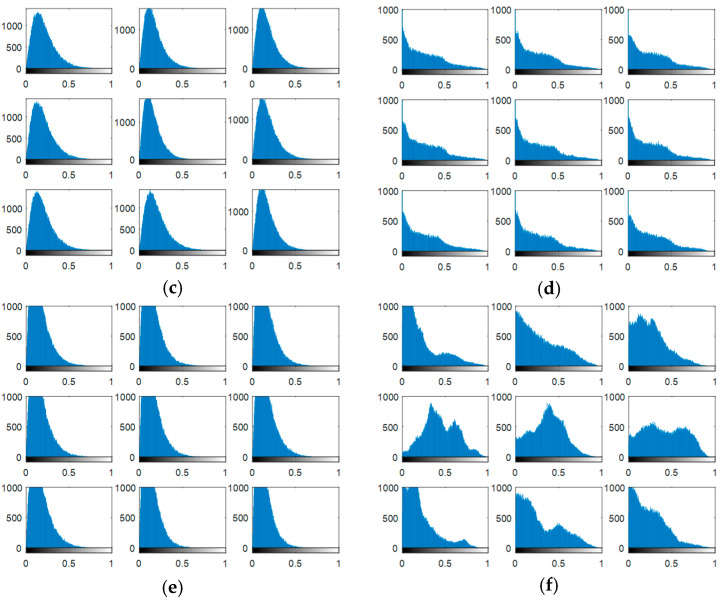
Histograms of encrypted images for the first database of the fingerprint biometrics. (**a**) Rotation in spatial domain; (**b**) Rotation followed by DWT; (**c**) Rotation in FFT domain; (**d**) Rotation in DCT domain; (**e**) Rotation in FrFT domain [45, 45]; (**f**) Rotation in FrFT domain [180, 90].

**Figure 19:**
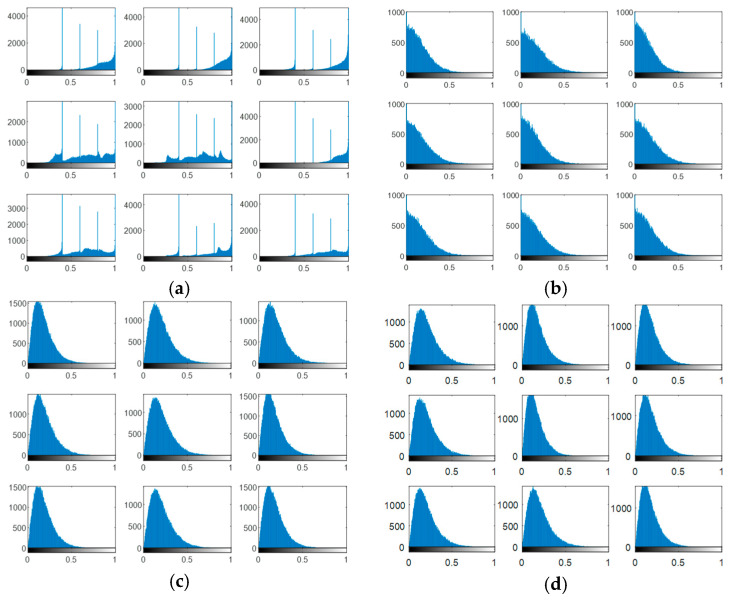

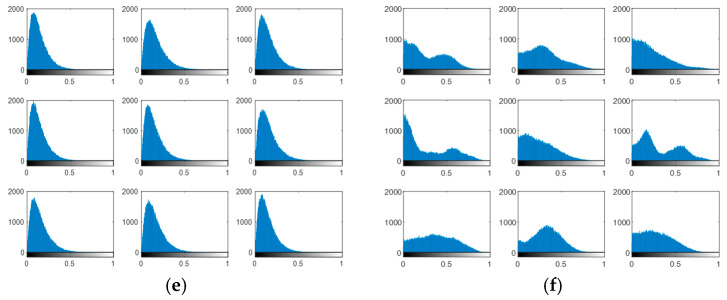
Histograms of encrypted images for the second database of the fingerprint biometrics. (**a**) Rotation in spatial domain; (**b**) Rotation followed by DWT; (**c**) Rotation in FFT domain; (**d**) Rotation in DCT domain; (**e**) Rotation in FrFT domain [45, 45]; (**f**) Rotation in FrFT domain [180, 90].

The output encrypted biometric images for faces and fingerprints are shown in Figure 10, Figure 11, Figure 12, Figure 13 and Figure 14 for the matrix rotations in spatial and different discrete domains. Figure 15, Figure 16, Figure 17, Figure 18 and Figure 19 illustrate the histograms of the face and fingerprint encrypted images. It is clear that original image histogram patterns are masked to a large extent. 

The efficiency of the proposed cancelable biometric methods is evaluated using the correlation coefficient and histogram between the protected biometrics stored in the database and their new biometric versions. The correlation coefficient can be measured as follows:

(14) $$R_{xy}=\frac{\frac{1}{N}\sum_{i=1}^{N}\left(x_{i}-\bar{x}\right)\left(y_{i}-\bar{y}\right)}{\sigma_{x}\sigma_{y}}$$

where *N* is the total number of pixels, *x* and *y* are the protected biometrics template in the database and the new issue protected template. 

**Figure 20:**
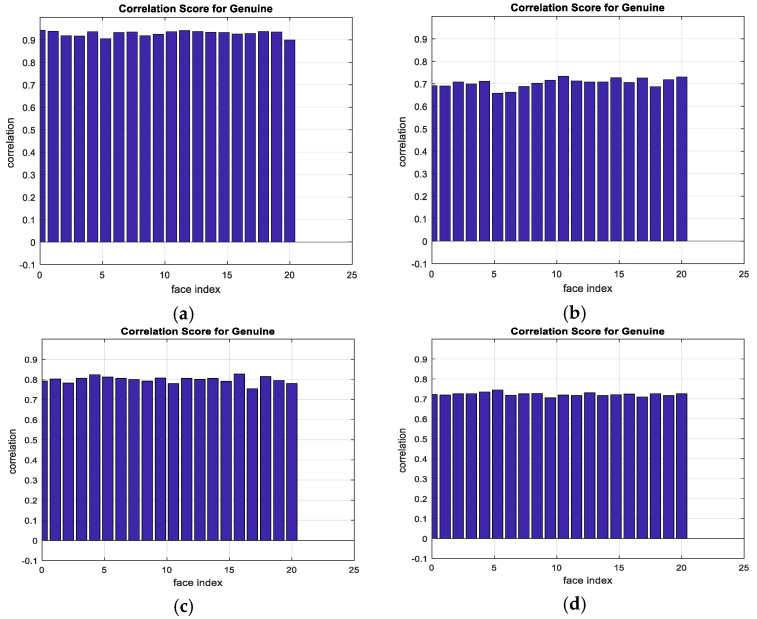

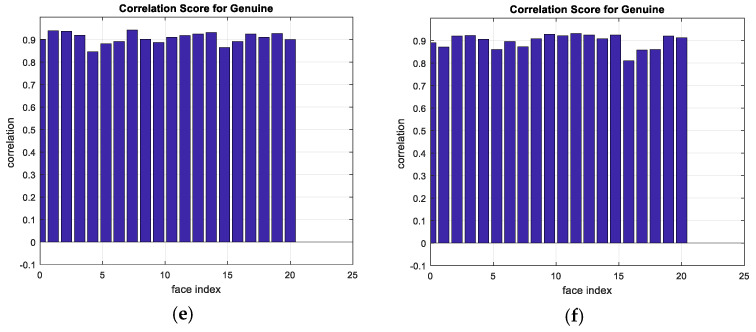
Correlation scores for authorized patterns of ORL face biometrics. (**a**) Rotation in spatial domain; (**b**) Rotation followed by DWT; (**c**) Rotation in FFT domain; (**d**) Rotation in DCT domain; (**e**) Rotation in FrFT domain [90, 90]; (**f**) Rotation in FrFT domain [370, 370].

**Figure 21:**
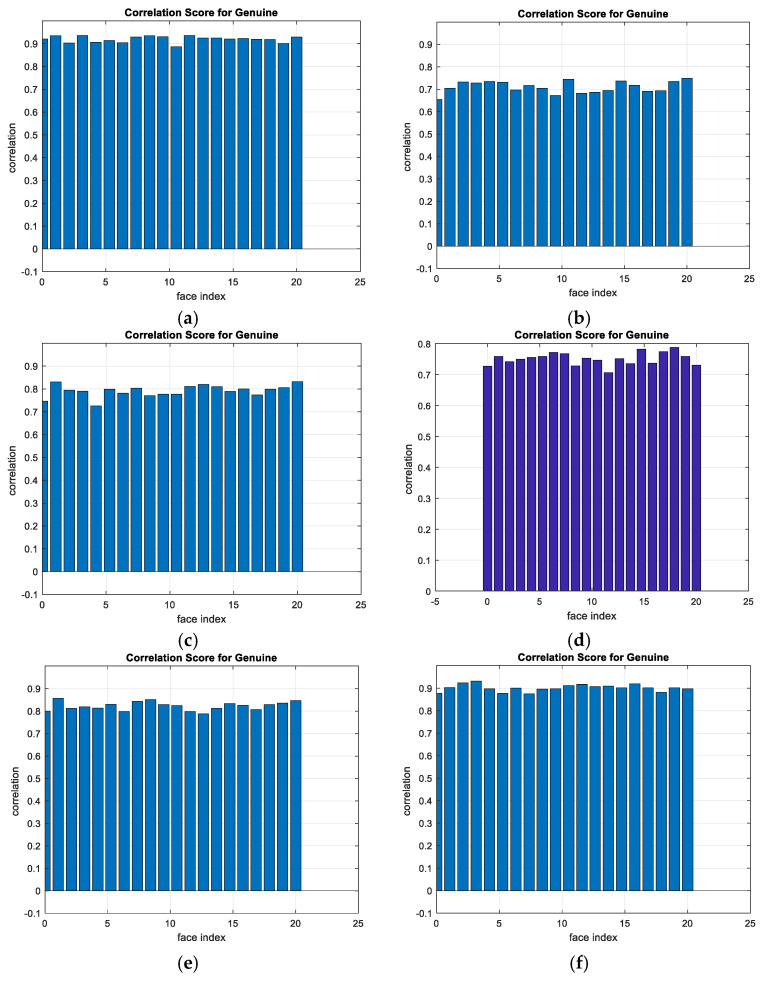
Correlation scores for authorized patterns of FERET face biometrics. (**a**) Rotation in spatial domain; (**b**) Rotation followed by DWT; (**c**) Rotation in FFT domain; (**d**) Rotation in DCT domain; (**e**) Rotation in FrFT domain [90, 90]; (**f**) Rotation in FrFT domain [370, 370].

**Figure 22:**
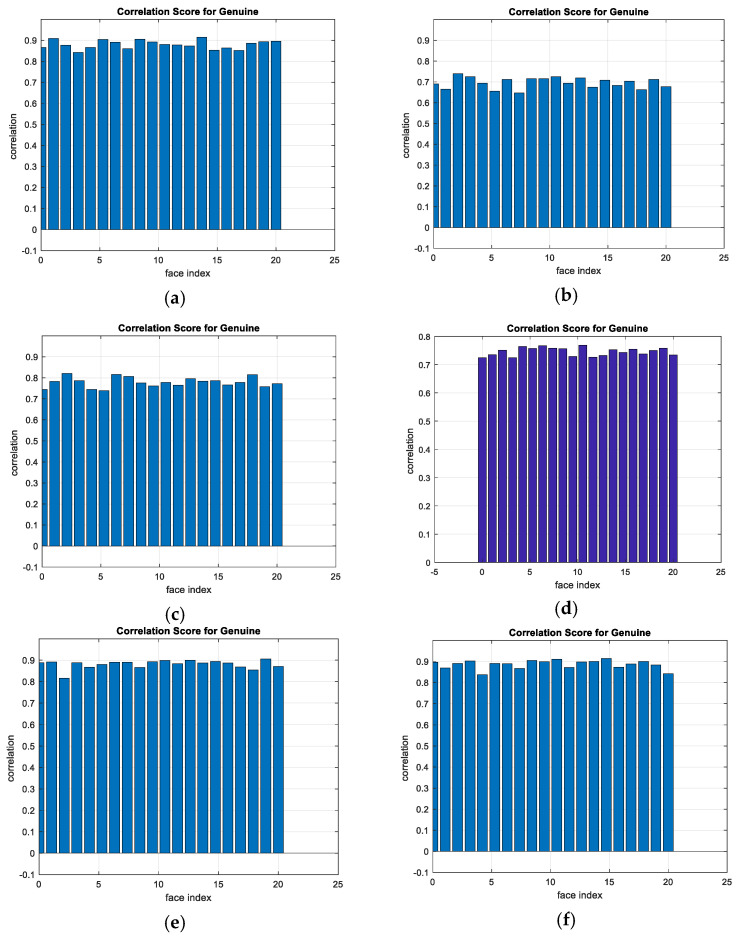
Correlation scores for authorized patterns of LFW face biometrics. (**a**) Rotation in spatial domain; (**b**) Rotation followed by DWT; (**c**) Rotation in FFT domain; (**d**) Rotation in DCT domain; (**e**) Rotation in FrFT domain [90, 90]; (**f**) Rotation in FrFT domain [370, 370].

**Figure 23:**
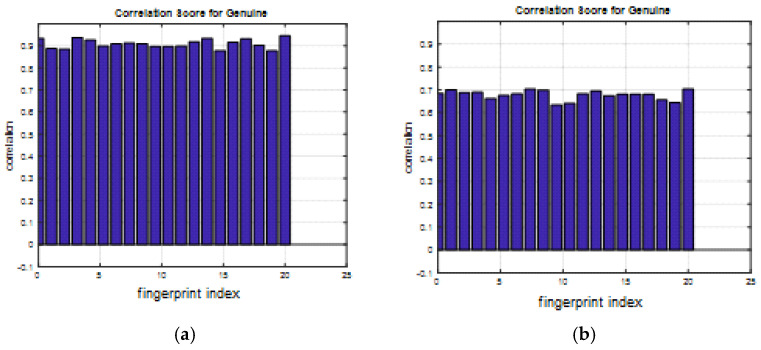

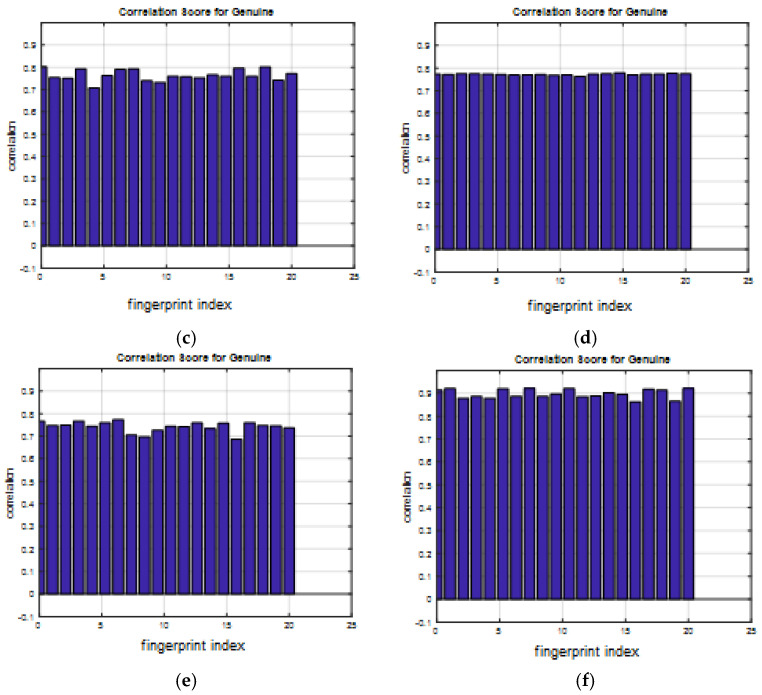
Correlation scores for authorized patterns of the first database of the fingerprint biometrics. (**a**) Rotation in spatial domain; (**b**) Rotation followed by DWT; (**c**) Rotation in FFT domain; (**d**) Rotation in DCT domain; (**e**) Rotation in FrFT domain [45, 45]; (**f**) Rotation in FrFT domain [180, 90].

**Figure 24:**
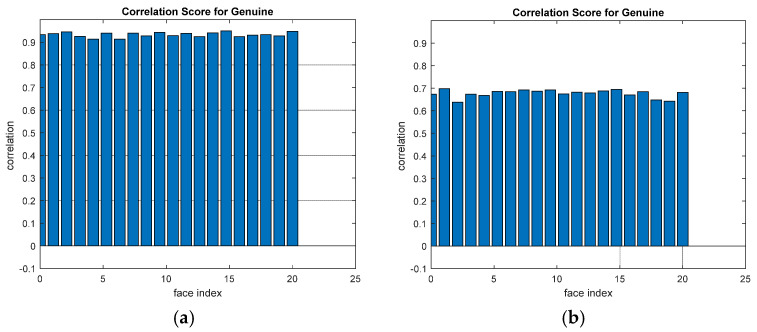

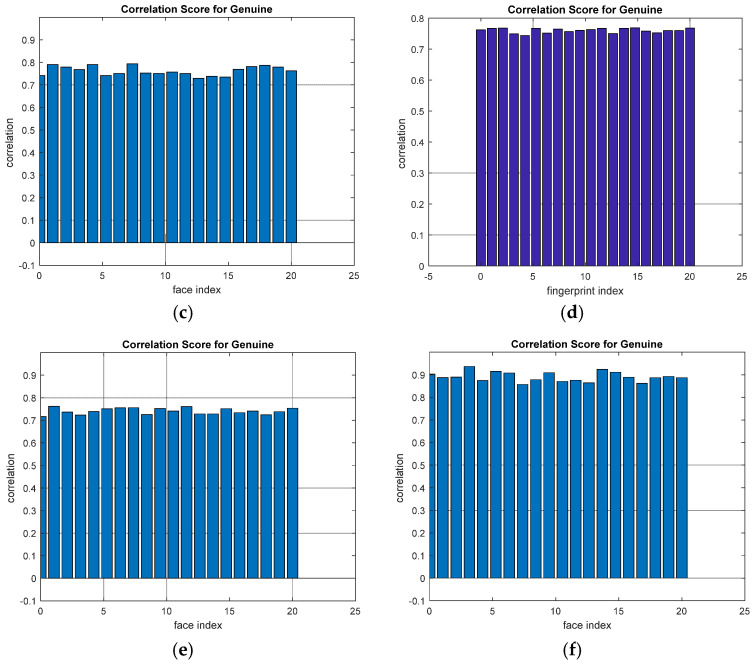
Correlation scores for authorized patterns of the second database of the fingerprint biometrics. (**a**) Rotation in spatial domain; (**b**) Rotation followed by DWT; (**c**) Rotation in FFT domain; (**d**) Rotation in DCT domain; (**e**) Rotation in FrFT domain [45, 45]; (**f**) Rotation in FrFT domain [180, 90].

**Figure 25:**
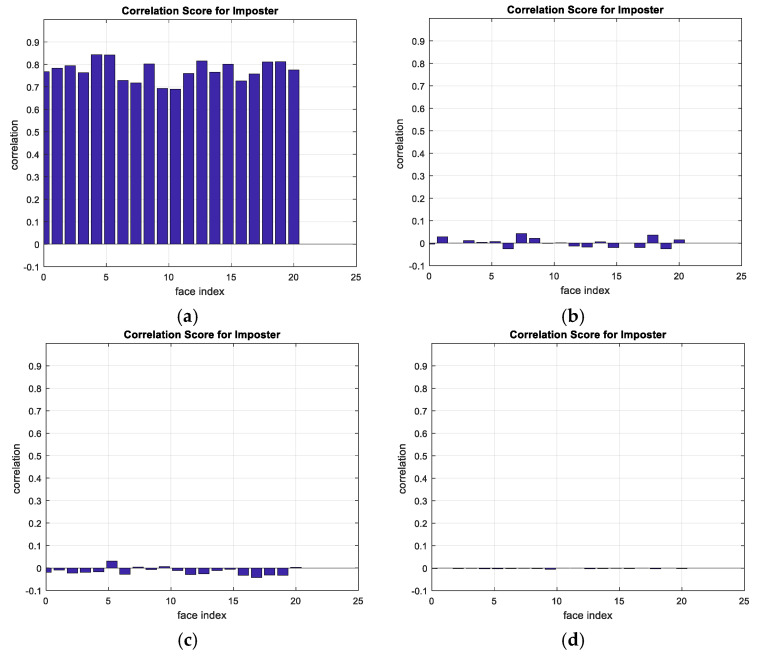

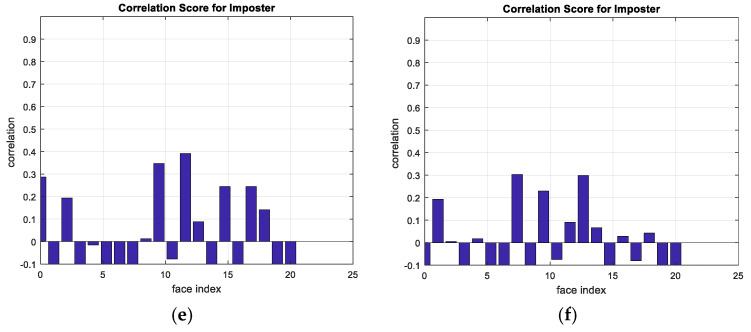
Correlation scores for unauthorized imposter patterns for ORL face biometrics. (**a**) Rotation in spatial domain; (**b**) Rotation followed by DWT; (**c**) Rotation in FFT domain; (**d**) Rotation in DCT domain; (**e**) Rotation in FrFT domain [90, 90]; (**f**) Rotation in FrFT domain [370, 370].

**Figure 26:**
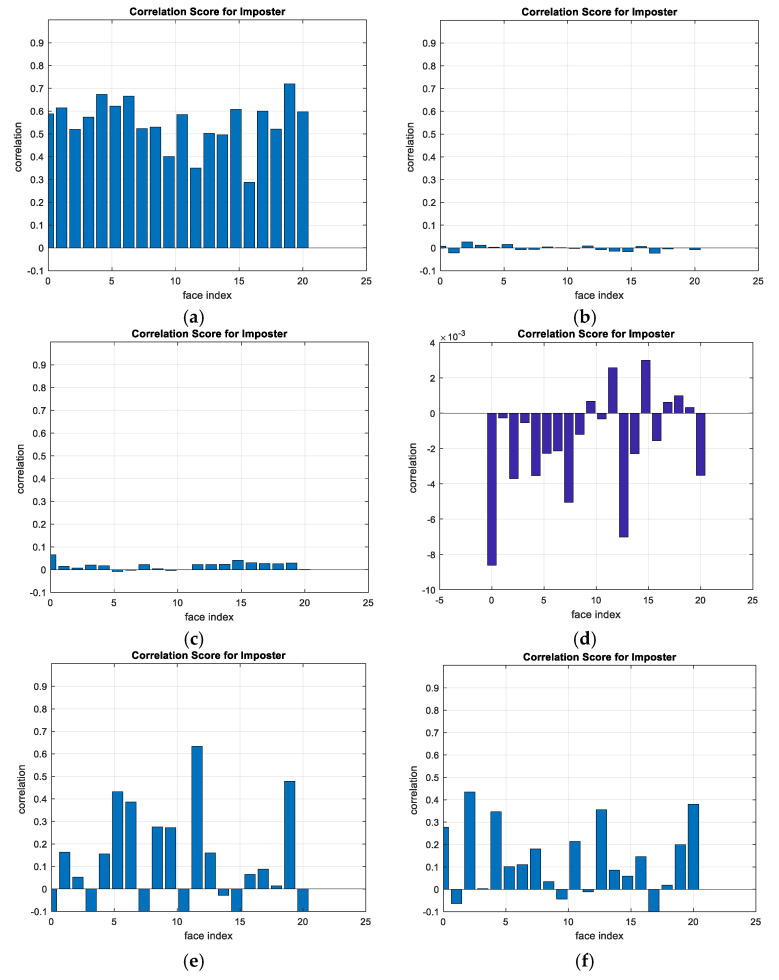
Correlation scores for unauthorized patterns of FERET face biometrics. (**a**) Rotation in spatial domain; (**b**) Rotation followed by DWT; (**c**) Rotation in FFT domain; (**d**) Rotation in DCT domain; (**e**) Rotation in FrFT domain [90, 90]; (**f**) Rotation in FrFT domain [370, 370].

**Figure 27:**
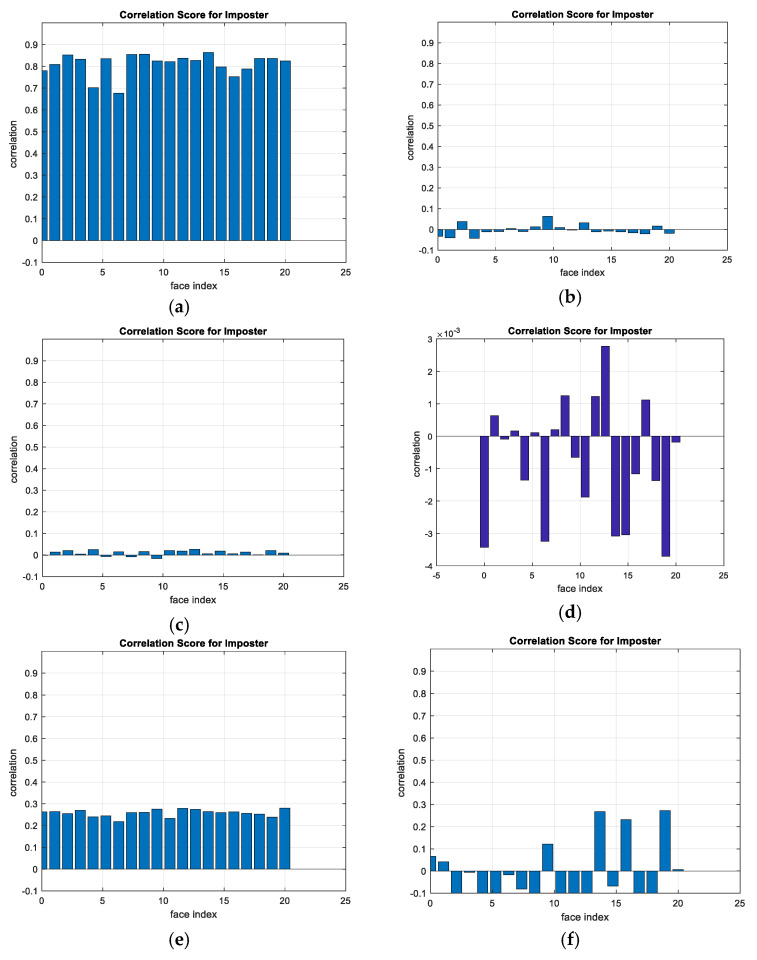
Correlation scores for unauthorized patterns of LFW face biometrics. (**a**) Rotation in spatial domain; (**b**) Rotation followed by DWT; (**c**) Rotation in FFT domain; (**d**) Rotation in DCT domain; (**e**) Rotation in FrFT domain [90, 90]; (**f**) Rotation in FrFT domain [370, 370].

**Figure 28:**
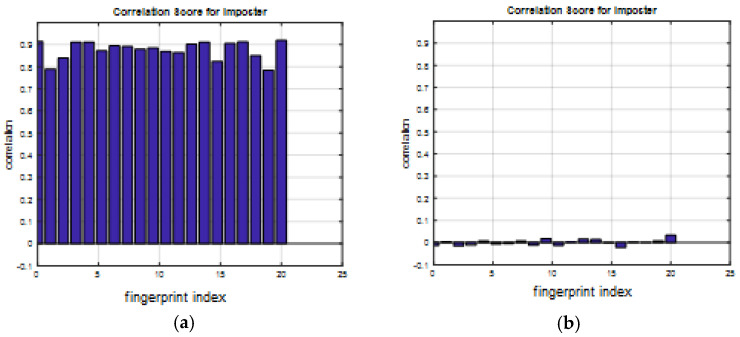

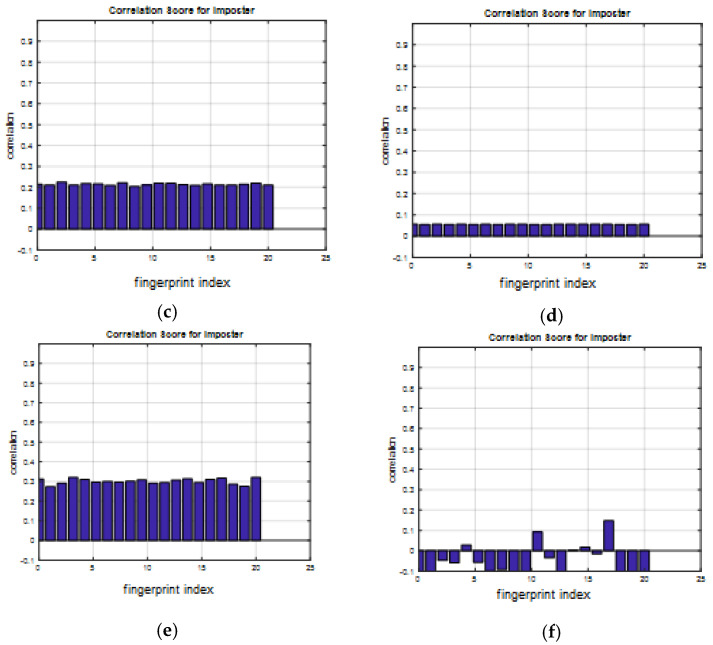
Correlation scores for unauthorized imposter patterns of the first database of the fingerprint biometrics. (**a**) Rotation in spatial domain; (**b**) Rotation followed by DWT; (**c**) Rotation in FFT domain; (**d**) Rotation in DCT domain; (**e**) Rotation in FrFT domain [45, 45]; (**f**) Rotation in FrFT domain [180, 90].

**Figure 29:**
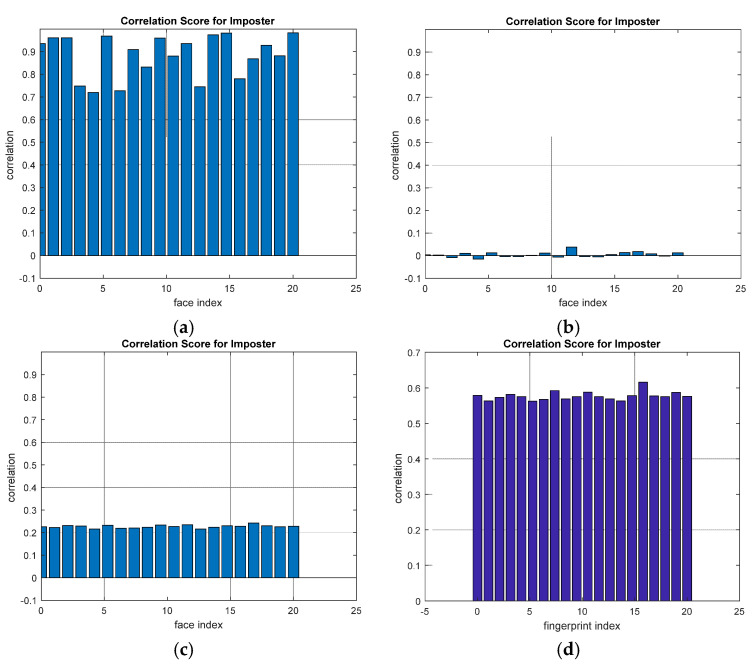

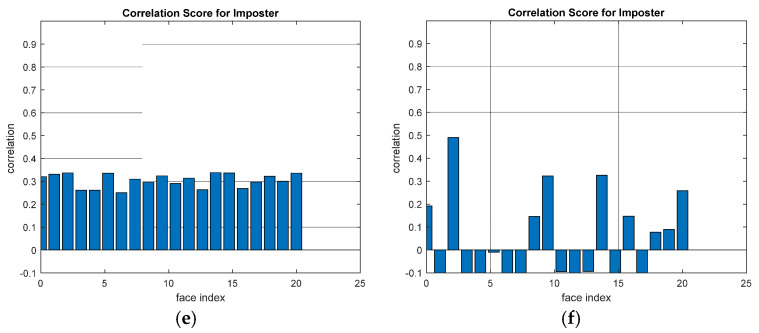
Correlation scores for unauthorized imposter patterns of the second database of the fingerprint biometrics. (**a**) Rotation in spatial domain; (**b**) Rotation followed by DWT; (**c**) Rotation in FFT domain; (**d**) Rotation in DCT domain; (**e**) Rotation in FrFT domain [45, 45]; (**f**) Rotation in FrFT domain [180, 90].

A comparison between the correlation scores for authorized patterns of the face and fingerprint patterns for the proposed methods is illustrated in Figure 20, Figure 21, Figure 22, Figure 23 and Figure 24, respectively. The correlation score is estimated between the tested genuine biometric and that stored in the database for all methods. Furthermore, a comparison of the correlation coefficient values estimated for unauthorized records with all records stored in the database is illustrated in Figure 25, Figure 26, Figure 27, Figure 28 and Figure 29 for face and fingerprint patterns, respectively. From this comparison, it is clear that the DCT-based method achieves the highest degree of security. 

**Figure 30:**
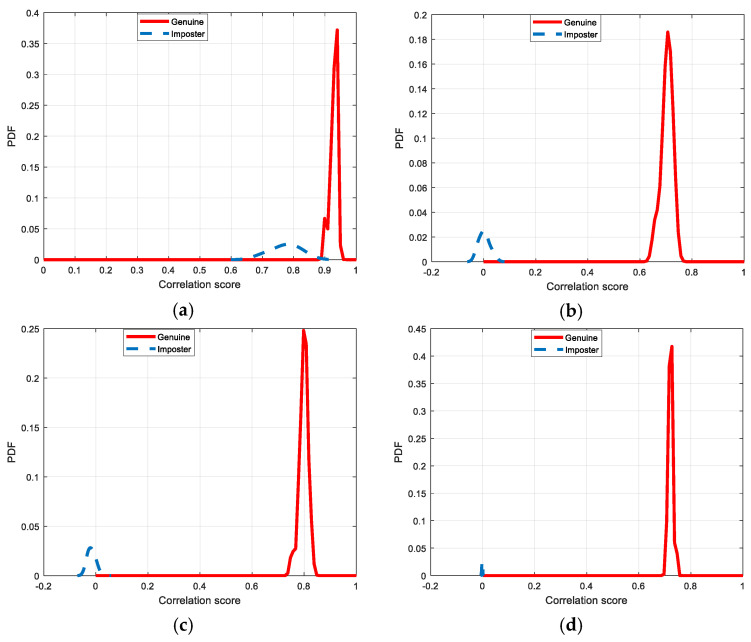

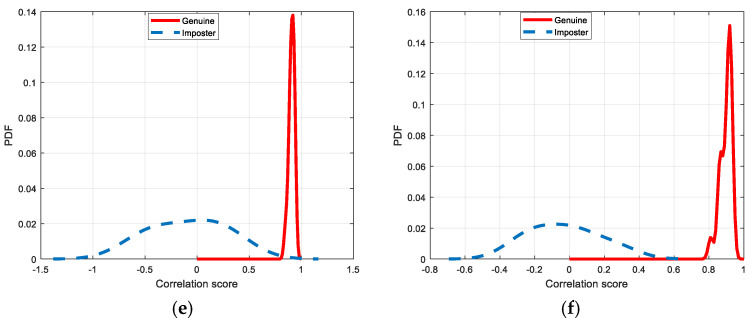
Probability Distributions Function (PDFs) for ORL face biometrics. (**a**) Rotation in spatial domain; (**b**) Rotation followed by DWT; (**c**) Rotation in FFT domain; (**d**) Rotation in DCT domain; (**e**) Rotation in FrFT domain [90, 90]; (**f**) Rotation in FrFT domain [370, 370].

**Figure 31:**
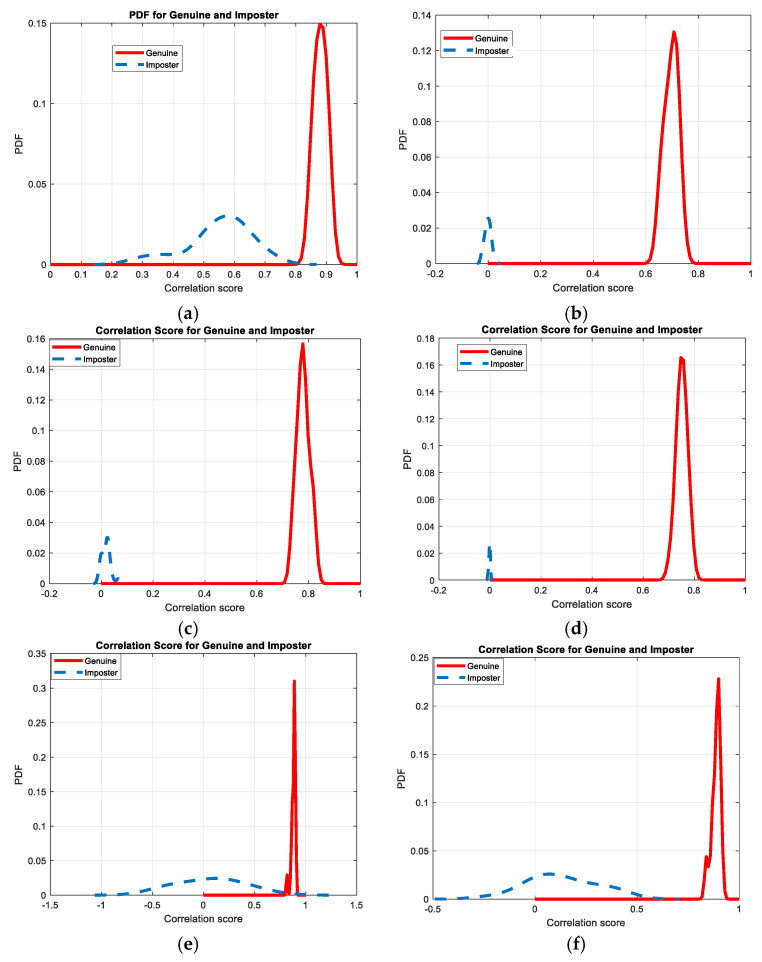
Probability Distributions Function (PDFs) for FERET face biometrics. (**a**) Rotation in spatial domain; (**b**) Rotation followed by DWT; (**c**) Rotation in FFT domain; (**d**) Rotation in DCT domain; (**e**) Rotation in FrFT domain [90, 90]; (**f**) Rotation in FrFT domain [370, 370].

**Figure 32:**
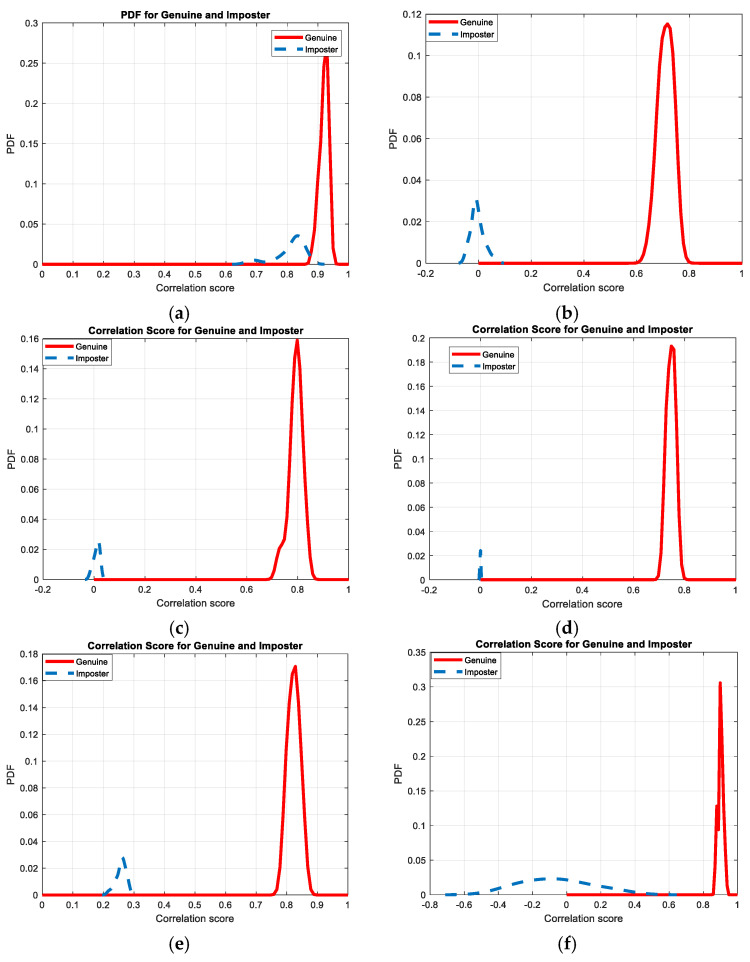
Probability Distributions Function (PDFs) for LFW face biometrics. (**a**) Rotation in spatial domain; (**b**) Rotation followed by DWT; (**c**) Rotation in FFT domain; (**d**) Rotation in DCT domain; (**e**) Rotation in FrFT domain [90, 90]; (**f**) Rotation in FrFT domain [370, 370].

**Figure 33:**
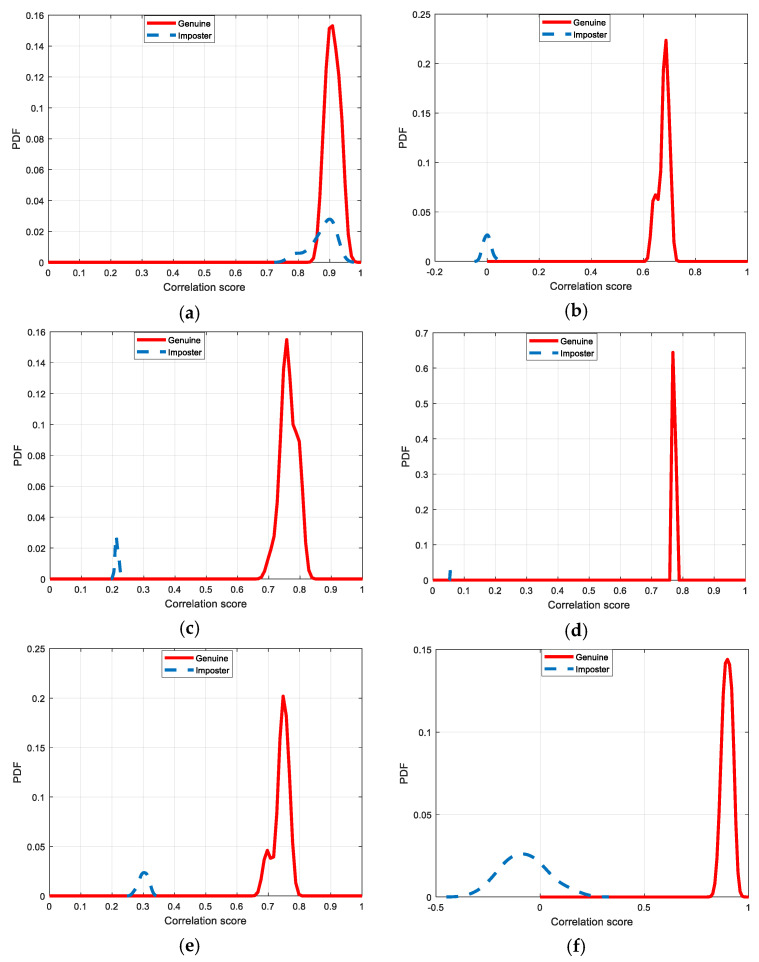
Probability Distributions Function (PDFs) for the first dataset of the fingerprint biometrics. (**a**) Rotation in spatial domain; (**b**) Rotation followed by DWT; (**c**) Rotation in FFT domain; (**d**) Rotation in DCT domain; (**e**) Rotation in FrFT domain [45, 45]; (**f**) Rotation in FrFT domain [180, 90].

**Figure 34:**
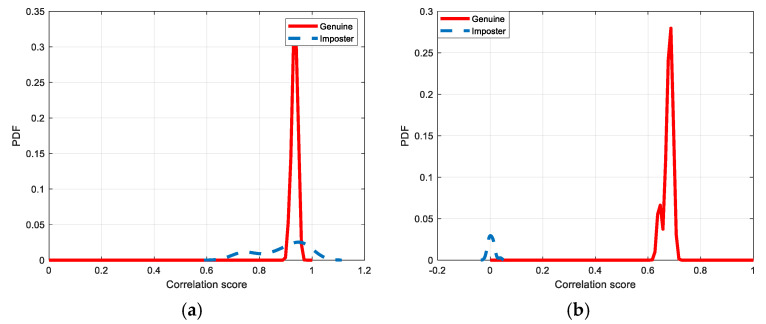

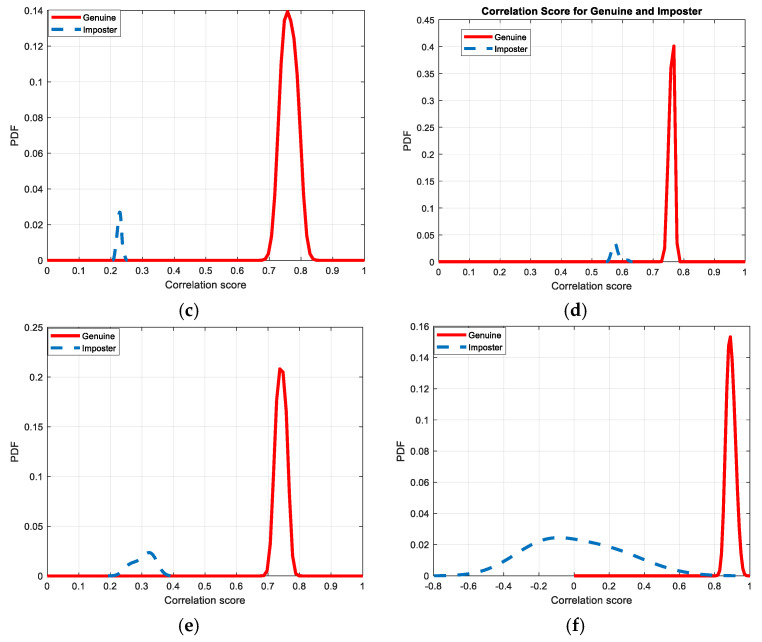
Probability Distributions Function (PDFs) for the second dataset of the fingerprint biometrics. (**a**) Rotation in spatial domain; (**b**) Rotation followed by DWT; (**c**) Rotation in FFT domain; (**d**) Rotation in DCT domain; (**e**) Rotation in FrFT domain [45, 45]; (**f**) Rotation in FrFT domain [180, 90].

**Figure 35:**
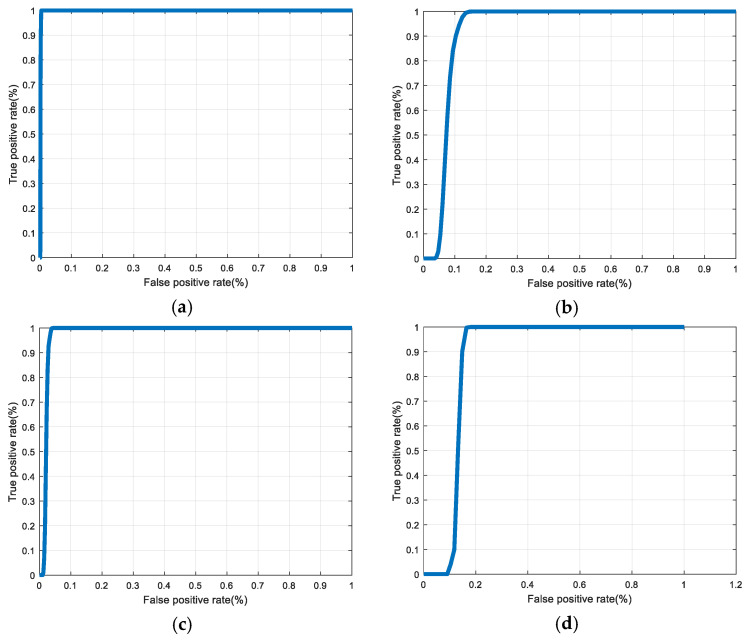

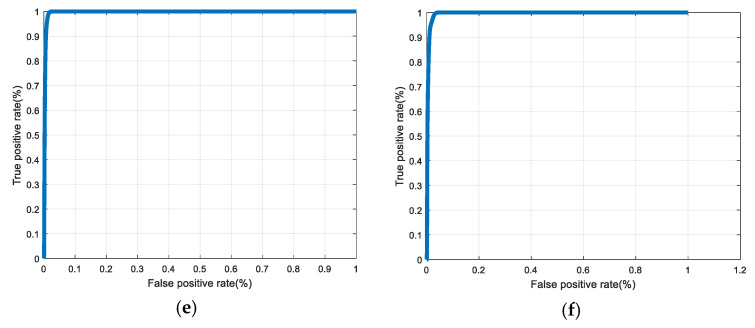
ROC curves for ORL face biometrics. (**a**) Rotation in spatial domain; (**b**) Rotation followed by DWT; (**c**) Rotation in FFT domain; (**d**) Rotation in DCT domain; (**e**) Rotation in FrFT domain [90, 90]; (**f**) Rotation in FrFT domain [370, 370].

**Figure 36:**
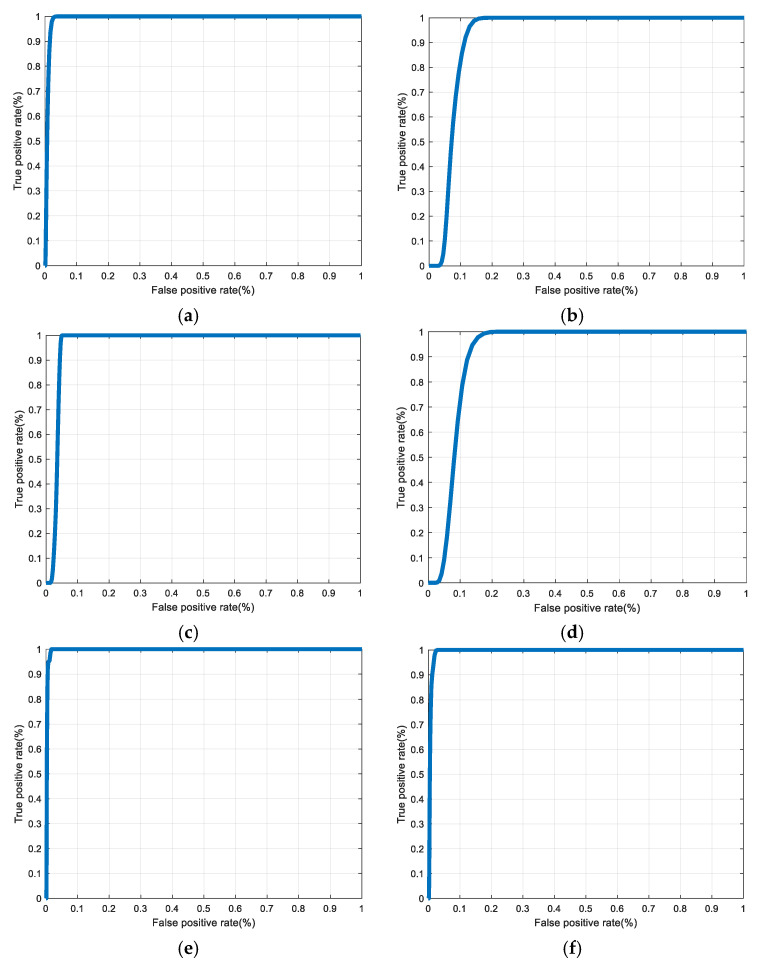
ROC curves for FERET face biometrics. (**a**) Rotation in spatial domain; (**b**) Rotation followed by DWT; (**c**) Rotation in FFT domain; (**d**) Rotation in DCT domain; (**e**) Rotation in FrFT domain [90, 90]; (**f**) Rotation in FrFT domain [370, 370].

**Figure 37:**
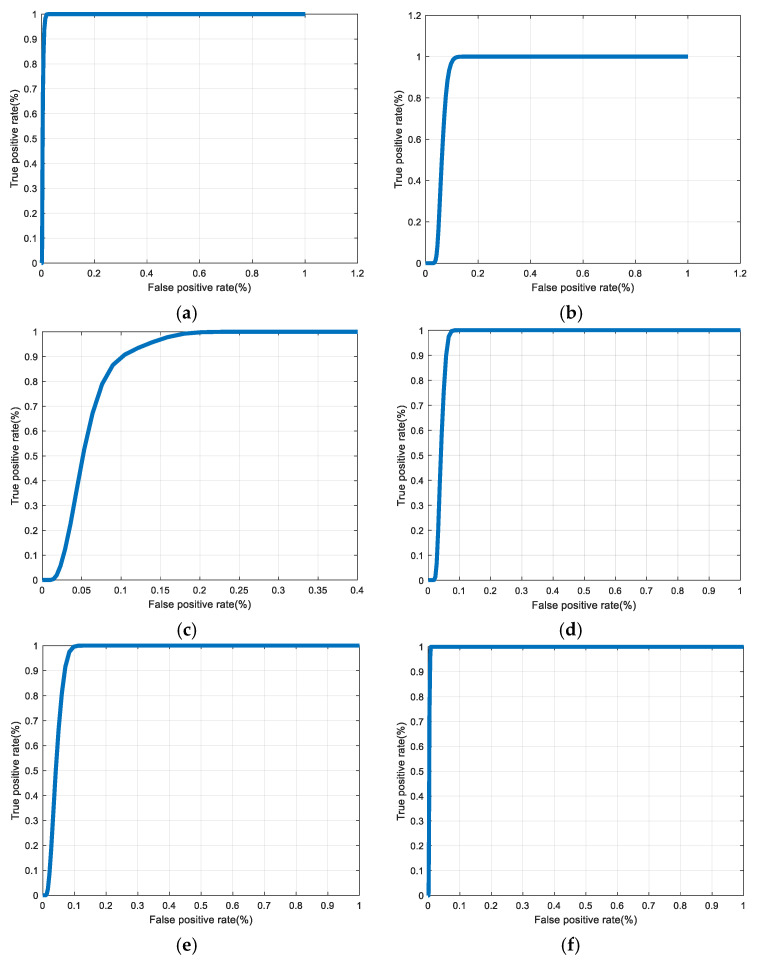
ROC curves for LFW face biometrics. (**a**) Rotation in spatial domain; (**b**) Rotation followed by DWT; (**c**) Rotation in FFT domain; (**d**) Rotation in DCT domain; (**e**) Rotation in FrFT domain [90, 90]; (**f**) Rotation in FrFT domain [370, 370].

**Figure 38:**
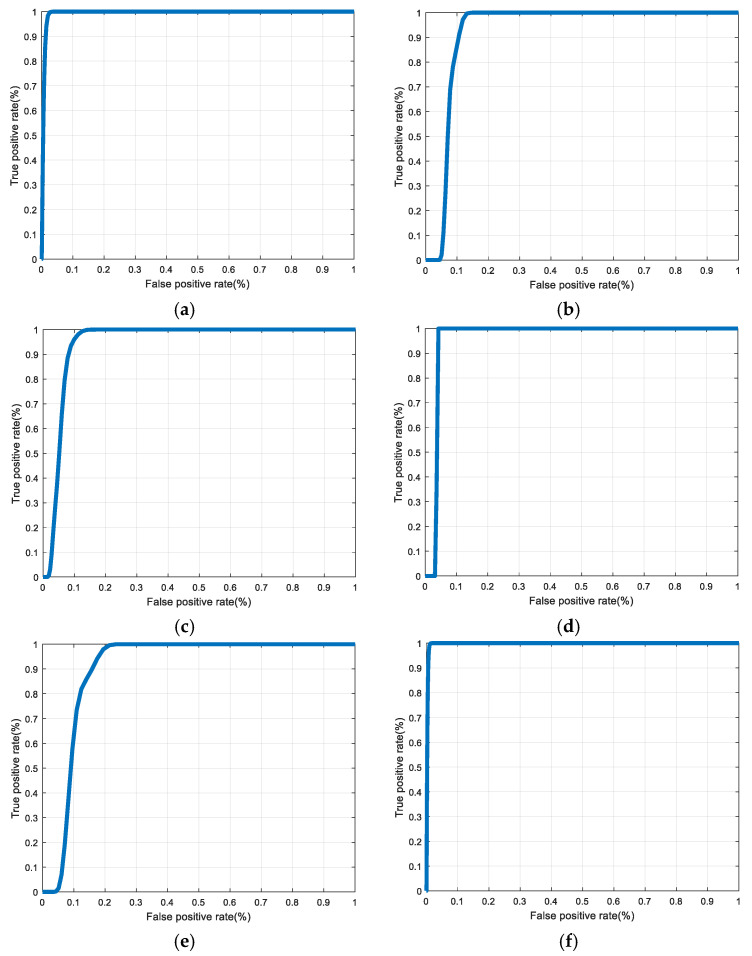
ROC curves for the first dataset of the fingerprint biometrics. (**a**) Rotation in spatial domain; (**b**) Rotation followed by DWT; (**c**) Rotation in FFT domain; (**d**) Rotation in DCT domain; (**e**) Rotation in FrFT domain [45, 45]; (**f**) Rotation in FrFT domain [180, 90].

**Figure 39:**
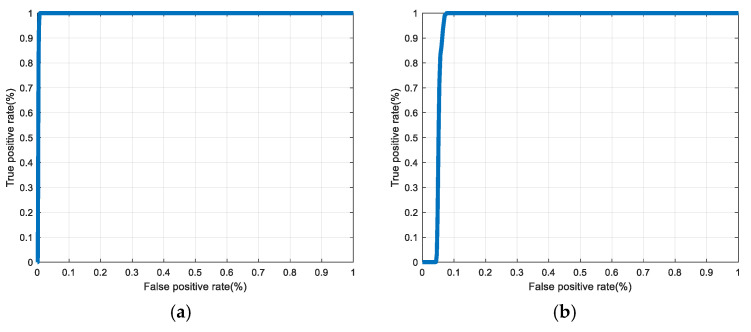

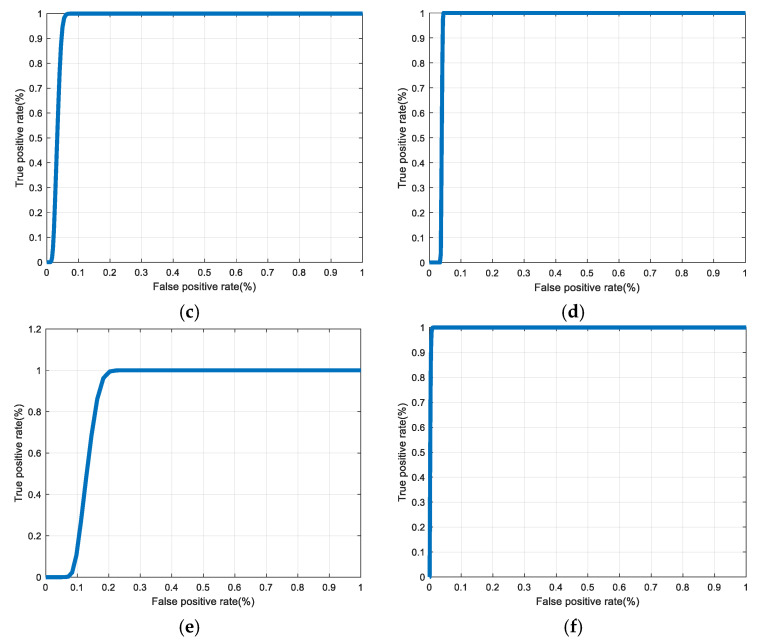
ROC curves for the second dataset of the fingerprint biometrics. (**a**) Rotation in spatial domain; (**b**) Rotation followed by DWT; (**c**) Rotation in FFT domain; (**d**) Rotation in DCT domain; (**e**) Rotation in FrFT domain [45, 45]; (**f**) Rotation in FrFT domain [180, 90].

Figure 30, Figure 31, Figure 32, Figure 33 and Figure 34 illustrate the probability distribution functions (PDFs) of the correlation coefficients in the genuine and imposter tests for the face and fingerprint biometrics. It can be noted that the two PDFs for genuine and imposter are distinctive except for the case of spatial-domain processing. The receiver operating characteristic (ROC) curves of the proposed methods for face and fingerprint biometrics are depicted in Figure 35, Figure 36, Figure 37, Figure 38 and Figure 39. As shown in the obtained results, the performance of rotation in the frequency domain using either the FFT or DFrFt is better than those of rotation in the DCT or DWT domain for face biometric datasets. Furthermore, the obtained results illustrate that the ROC plots of the DFrFT-based method that gives the best results at [90, 180], which confirms the superiority of the proposed rotation-based method in DFrFT domain and the effect of the angle “α” on the obtained results.

A comparative study between AROC values, mean of authorized correlation sores, mean of unauthorized correlation sores, FAR, FRR, and ERR for all proposed cancelable biometric methods is presented. The results of this comparison are tabulated in Table 1, Table 2, Table 3, Table 4 and Table 5 for the face and fingerprint datasets. The results reveal that the rotation in the DFrFT-based method achieves a high level of security for face and finger biometric templates.

To supplementary substantiate the effectiveness of the suggested cancelable biometric recognition method, test experimentations have been employed for comparing outcomes of the suggested cancellable biometric recognition method with those of the recent previous methods [19,31,34,51,52,53,54,55]. We compared the average EER, FAR, FRR, and AROC results of the suggested method with those of the methods in [19,31,34,51,52,53,54,55] as given in Table 6. Form the offered comparative outcomes in Table 6, we observe that the FRR, FAR, AROC, and EER outcomes of the suggested method are more recommended and appreciated contrasted to those of the other conventional methods.

## 5. Conclusions and Future Work

This paper dealt with the generation of cancelable biometric templates with sophisticated methods based on discrete transforms and matrix rotations. Two types of biometrics were processed with the proposed methods: faces and fingerprints. The main objective of the suggested methods is to generate cancelable templates with as simple algorithms as possible and avoid high-complexity encryption schemes. The diffusion characteristic of most discrete transforms is exploited to tailor a pattern upon which we can depend for the generation of the cancelable templates. In fact, these transforms are not enough as they are invertible. Hence, rotation is exploited. A single rotation is not a feasible action as it can be inverted easily. The solution suggested is to use multiple rotations, and hence an addition process of rotated versions. This process is irreversible, which is the characteristic that guarantees the security of original biometric templates. In addition, there is a freedom in selecting the number of rotations, and rotation angles to allow generation of multiple templates for different applications and for hacking scenarios. Simple rotations are not time-consuming, and hence the suggested methods have low complexity. Different discrete transforms have been exploited and compared in the proposed methods, including DWT, DCT, FFT, and FrFT transforms. Cancelability is tested and evaluated through extensive simulation results for all proposed methods. Low EER values with high AROC values reflect the efficiency of the proposed methods, especially those dependent on DCT and DFrFT. For future plans, we can test other different discrete transforms in the Quaternion domain by incorporating encryption, watermarking, and steganography techniques for building efficient cancelable biometric recognition systems.

## Figures and Tables

**Table 1:** Comparative results of the recognition methods on the ORL faces dataset.

Method	AROC	Mean of Authorized Correlation Score	Mean of Un-Authorized Correlation Score	FAR	FRR	ERR
Cancelable based rotation	0.9986	0.9286	0.7725	0.0047	0.0024	0.0018
Rotation followed by DWT	0.9244	0.7041	0.0024	0.135	0.0228	0.0144
Rotation in FFT domain	0.9784	0.7984	−0.0152	0.0381	0.0209	0.0106
Rotation based on DCT	0.8681	0.7228	−0.0019	0.1641	0.1000	0.0568
Rotation based on FrFT [90, 90]	0.9969	0.907	−0.0897	0.0173	0.0112	0.0055
Rotation based on FrFT [180, 180]	0.9967	0.8968	−0.0092	0.0125	0.0104	0.005
Rotation based on FrFT [370, 370]	0.9952	0.8976	−0.0320	0.0311	0.0149	0.0072

**Table 2:** Comparative results of the recognition methods on the FERET faces dataset.

Method	AROC	Mean of Authorized Correlation Score	Mean of Un-Authorized Correlation Score	FAR	FRR	ERR
Cancelable based rotation	0.9920	0.8802	0.5490	0.0266	0.0212	0.0107
Rotation followed by DWT	0.9236	0.6961	−0.0012	0.1424	0.0354	0.0184
Rotation in FFT domain	0.9657	0.7788	0.0182	0.0497	0.0075	0.0042
Rotation based on DCT	0.914	0.751	−0.0017	0.1559	0.0536	0.0245
Rotation based on FrFT [90, 90]	0.9965	0.8810	0.0628	0.0171	0.0159	0.0091
Rotation based on FrFT [180, 180]	0.9964	0.8971	−0.0087	0.0159	0.0130	0.007
Rotation based on FrFT [370, 370]	0.9941	0.8864	0.1304	0.0238	0.0220	0.0120

**Table 3:** Comparative results of the recognition methods on the LFW faces dataset.

Method	AROC	Mean of Authorized Correlation Score	Mean of Un-Authorized Correlation Score	FAR	FRR	ERR
Cancelable based rotation	0.9953	0.9199	0.8104	0.0190	0.0182	0.0109
Rotation followed by DWT	0.9363	0.7103	−0.0032	0.1118	0.0161	0.0088
Rotation in FFT domain	0.9404	0.7921	0.0100	0.1592	0.0419	0.0201
Rotation based on DCT	0.9581	0.7462	−0.0007	0.0749	0.0258	0.0158
Rotation based on FrFT [90, 90]	0.9561	0.8231	0.2578	0.0973	0.0254	0.0172
Rotation based on FrFT [180, 180]	0.9965	0.8966	−0.0089	0.0131	0.0114	0.008
Rotation based on FrFT [370, 370]	0.9966	0.9015	0.0616	0.0081	0.0418	0.0213

**Table 4:** Comparative results of the recognition methods on the first fingerprint database.

Method	AROC	Mean of Authorized Correlation Score	Mean of Un-Authorized Correlation Score	FAR	FRR	ERR
Cancelable based rotation	0.993	0.91	0.876	0.026	0.017	0.010
Rotation followed by DWT	0.925	0.6777	0.0004	0.131	0.028	0.0187
Rotation in FFT domain	0.953	0.7699	0.2135	0.104	0.014	0.0130
Rotation based on DCT	0.963	0.772	0.0552	0.042	0.644	0.3251
Rotation based on FrFT [45, 45]	0.901	0.7420	0.3009	0.194	0.057	0.0278
Rotation based on FrFT [180, 180]	0.991	0.8879	0.0521	0.039	0.014	0.0076
Rotation based on FrFT [180, 90]	0.997	0.8980	−0.0751	0.012	0.010	0.0052

**Table 5:** Comparative results of the recognition methods on the second fingerprint database.

Method	AROC	Mean of Authorized Correlation Score	Mean of Un-Authorized Correlation Score	FAR	FRR	ERR
Cancelable based rotation	0.9974	0.9338	0.8841	0.008	0.003	0.0030
Rotation followed by DWT	0.9474	0.6771	0.0042	0.076	0.010	0.0081
Rotation in FFT domain	0.9667	0.7623	0.2271	0.060	0.018	0.0097
Rotation based on DCT	0.9608	0.7596	0.5771	0.044	0.035	0.0183
Rotation based on FrFT [45, 45]	0.8683	0.7412	0.3049	0.203	0.038	0.0264
Rotation based on FrFT [180, 180]	0.9909	0.8882	0.0531	0.037	0.015	0.0072
Rotation based on FrFT [180, 90]	0.997	0.8913	0.0120	0.010	0.003	0.0026

**Table 6:** The average statistical evaluation results for the proposed and traditional cancellable biometric methods [19,31,34,51,52,53,54,55].

Cancellable Biometric Method	EER	FAR	FRR	AROC
Proposed	0.0023	0.008	0.003	0.998
Ref. [19]	0.0924	0.0562	0.0257	0.868
Ref. [31]	0.0178	0.0071	0.0876	0.896
Ref. [34]	0.0098	0.0104	0.018	0.952
Ref. [51]	0.1081	0.0927	0.0967	0.907
Ref. [52]	0.0416	0.1955	0.0489	0.873
Ref. [53]	0.0859	0.0435	0.0627	0.718
Ref. [54]	0.0357	0.0985	0.0612	0.863
Ref. [55]	0.0046	0.0235	0.0929	0.883
